# Structures, photoresponse properties and DNA binding abilities of 4-(4-pyridinyl)-2-pyridone salts†

**DOI:** 10.1039/c9ra00666d

**Published:** 2019-03-27

**Authors:** Tripti Mandal, Arka Dey, Sudipta Pathak, Md. Maidul Islam, Saugata Konar, Joaquín Ortega-Castro, Saikat Kumar Seth, Partha Pratim Ray, Antonio Frontera, Subrata Mukhopadhyay

**Affiliations:** Department of Chemistry, Jadavpur University Jadavpur Kolkata 700032 India; Department of Condensed Matter Physics and Material Sciences, S. N. Bose National Centre for Basic Sciences Block JD, Sec. III, Salt Lake Kolkata 700106 India; Department of Physics, Jadavpur University Jadavpur Kolkata 700032 India skseth@phys.jdvu.ac.in ray.parthapratim@gmail.com; Department of Chemistry, Haldia Government College Debhog, Purba Medinipur West Bengal 721657 India; Department of Chemistry, Aliah University Action Area IIA/27 Kolkata 700156 India; Department of Chemistry, Bhawanipur Education Society College Bhowanipore Kolkata 700020 India; Departament de Química, Universitat de les Illes Balears Crta. de Valldemossa km 7.5 07122 Palmade Mallorca Baleares Spain toni.frontera@uib.es

## Abstract

Three salts [perchlorate (2), chloride (3) and tetrafluoroborate (4)] were synthesized from a 1-(2-aminoethyl)-6-hydroxy-2-oxo-1,2-dihydro-[4,4′-bipyridine]-3,5-dicarbonitrile compound (1) and characterized by spectroscopic and single crystal X-ray diffraction methods. Various noncovalent interactions (*e.g.*, anion⋯π^+^, π⋯π, lp⋯π) are explored in the solid state crystal structure of the salts. Optical band gaps of all the four compounds were determined from their solid-state UV-vis spectrum. Electrical properties like electrical conductivity, photosensitivity, *etc.* were calculated and the results revealed that they have potential to act as optoelectronic devices. The values of the electrical parameters increase several times when they are exposed to visible light rather than in dark conditions. The light sensing properties of the salts (2–4) are enhanced compared to that of the mother organic compound 1 but the magnitude of this enhancement is not same for the three salts. This observation has been rationalized by theoretical considerations. Moreover, the DNA binding ability of one of the representative salts (compound 2) was examined to check the biological importance of the synthesized salts.

## Abstract 2

The optical band gap energies in several 2-pyridone derivatives have been measured using solid state UV to explore their semiconductor behavior. The electric current measurements for the four compounds exhibit enhanced photoconduction properties under irradiation of light.

## Graphical abstract

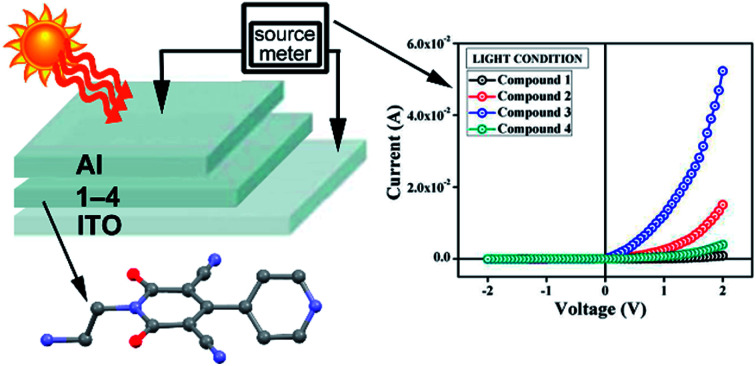

## 1. Introduction

The synthesis and study of materials that are able to exhibit semiconducting behaviour has already gained considerable attention in recent years for their potential applications in electronic devices. It is one of the technology-driven research areas that is becoming attractive to engineers, scientists and the relevant people including the policy makers. Such organic semiconductors possess a number of advantages over conventional inorganic semiconductors like light weight and flexibility as well as their films being easy to deposit over large areas.^1–6^ The application spectrum of these organic based semiconductors include sensors, organic light-emitting diodes (OLEDs), organic field effect transistors (OFETs), memory devices, photoconductors, generation of electricity from solar energy, diodes of radio frequency identification tags and many more.^7–14^ The prolific way to control and enhance electrical conductivity of such organic materials is through extended π-conjugation^15,16^ or introducing an ionic character by salt formation.^17,18^ Organic compounds having protonation sites can improve their electronic properties many times over by protonation induced modification of the organic semiconductors.^19^ In some polyaniline compounds, the leucoemeraldine base form is almost an insulator with a large band gap (∼3.6 eV) whereas the emeraldine salt form shows a band gap of 1.5 eV from experimental absorption spectrum.^20^ Moreover, the relative orientation of molecular constituents significantly controls the magnitude and efficiency of charge transport of semiconducting materials in the solid-state.^21–26^ Preferred orientation of molecular constituents increases π electron conjugation and also the semiconducting property of organic molecules. In the case of a biphenyl system which is very close to our synthesized compounds, a molecule with two phenyl rings linked by a single C–C bond, conductance is expected to change with the relative twist angle between the two rings; the planar conformation is expected to have the highest conductance.^27^ Till now semiconducting properties due to the presence of extended π-conjugation in organic polymers have been utilized^28–34^ to fabricate optoelectronic devices, but less attention has been paid to explore such properties in organic salts.^35^ Moreover, exploration of weak interactions expected to be present in the solid state structures of the organic salts with aromatic rings^36–40^ is another objective of the present study.

**Fig. 1:**
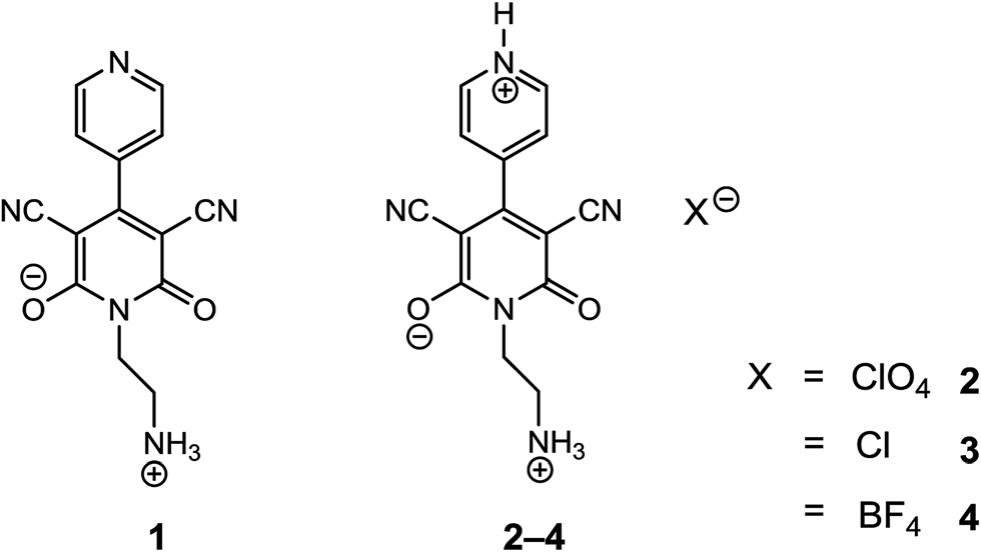
Structures of compounds 1–4.

In the present work, we report preparations of three different salts [perchlorate (2), chloride (3) and tetrafluoroborate (4)] of 1-(2-aminoethyl)-6-hydroxy-2-oxo-1,2-dihydro-[4,4′-bipyridine]-3,5-dicarbonitrile (Fig. 1, compound 1) with a common cationic 4-(4-pyridinyl)-2-pyridone moiety and establish their solid-state structures. Moreover, we have analysed their photoresponse properties since all of them (including compound 1) show optical band gaps in the semiconductor range. It is noteworthy to mention that we noticed remarkable differences in optical and semiconducting properties in 1–4. We have also rationalized the semiconductor and photoresponse behaviour by means of theoretical calculations. Additionally, it may be noted that the 2-pyridone derivatives have biological importance because of their ability to be used as antimalarial, antitumoral, analgesic and anti-HIV agents. More specifically 3-cyano-2-pyridone frameworks has been used before for the treatment of congestive heart failure and for anticancer activity, PIM1 kinase inhibitor and survivin protein.^41–44^ In order to explore more about the biological importance of 2-pyridone derivatives, we examined the DNA binding ability of a representative salt (2) in aqueous buffer medium as salt formation (cation) can improve bioavailability of a drug through chemical approaches without changing the active target.^45^ Molecular docking and circular dichroic study has also been carried out to support the experimental results.

## 2. Experimental

### 2.1. Materials and measurements

All chemicals used were purchased from Sigma-Aldrich Chemical Co. and used without further purification. All reactions were carried out in aerobic conditions and in aqueous medium. Freshly boiled, doubly distilled water was used when necessary. IR spectra were recorded on a Perkin-Elmer RXI FT-IR spectrophotometer with the sample prepared as a KBr pellet, in the range 4000–400 cm^−1^. Elemental analyses (C, H, N) were performed on a Perkin-Elmer 240C elemental analyser. The PXRD data of the powdered sample were collected on a Rigaku-TTRAX-III diffractometer using Cu Kα radiation (*λ* = 1.5406 Å). Thermogravimetric analysis (TGA) was performed on a Perkin-Elmer Thermal Analyzer TGA 4000 in a broad temperature range between 30 °C and 850 °C under nitrogen atmosphere.

### 2.2. Synthesis of [HAHPD]ClO_4_·2H_2_O (2)

The organic compound AHPD [AHPD = 1-(2-aminoethyl)-6-hydroxy-2-oxo-[4,4′-bipyridine]-1,2-dihydropyridine-3,5-dicarbonytriles] (1) was prepared and characterized following the literature method.^46^ Aqueous suspension of 1 (0.5 mM, 0.140 g) was reacted with dilute HClO_4_ at room temperature (∼25.0 °C) with continuous stirring until a clear solution was obtained (pH ∼ 0.5). The resultant solution was then filtered and left unperturbed for crystallization. After a few days, block shaped, yellow single crystals of 2 (0.132 g, 63%) suitable for X-ray analysis were obtained. The crystals were collected by filtration, washed with cold water, and dried in air. Anal. calcd for C_14_H_16_ClN_5_O_8_ (2): C, 40.25; H, 3.86; N, 16.76%. Found: C, 40.39; H, 3.77; N, 16.83%. Main IR absorption bands observed for 2 (KBr pellet/cm^−1^) are 3102 (w), 2961 (w), 2206 (s), 1627 (s), 1521 (s), 1472 (s), 1091 (s), 774 (s), 626 (s), 505 (s).

### 2.3. Synthesis of [HAHPD]Cl (3)

Compound 3 was synthesized using the same procedure as above except aqueous solution of HCl was reacted to aqueous suspension of 1 (0.5 mM, 0.140 g) instead of aqueous HClO_4_ solution. After two weeks single crystals of 3 was obtained by slow evaporation of mother solution at room temperature and collected by filtration (0.106 g, 66.8%). Anal. calcd for C_14_H_12_ClN_5_O_2_ (3): C, 52.92; H, 3.81; N, 22.04%. Found: C, 52.81; H, 3.85; N, 22.17%. Main IR absorption bands observed for 3 (KBr pellet, cm^−1^) are 3384 (w), 3159 (w), 2777 (s), 2721 (s), 2488 (s), 2206 (s), 1564 (s), 1514 (s), 1465 (s), 1211 (s), 844 (s), 775 (s), 612 (s), 499 (s), 414 (s).

### 2.4. Synthesis of [HAHPD]BF_4_·2H_2_O (4)

Aqueous suspension of 1 (0.5 mM, 0.140 g) was reacted with aqueous HBF_4_ solution (45% in water) to obtain clear solution (pH ∼ 0.5). Mother solution was filtered and left unperturbed for slow evaporation at room temperature. After 15 days, block shaped, yellow single crystals of 4 (0.117 g, 58%) suitable for X-ray analysis were collected from mother solution. Anal. calcd for C_14_H_16_BF_4_N_5_O_4_ (4): C, 41.51; H, 3.98; N, 17.29%. Found: C, 41.37; H, 3.89; N, 17.39%. Main IR absorption bands observed for 4 (KBr pellet, cm^−1^) are 3582 (s), 3222 (s), 3081 (w), 2954 (w), 2206 (s), 1620 (s), 1571 (s), 1522 (s), 1472 (s), 1056 (w), 1000 (w), 767 (s), 513 (s).

### 2.5. X-ray crystallography study

Single crystal X-ray diffraction intensity data of compounds 2–4 were collected at 293(2) K using a Bruker APEX-II CCD diffractometer equipped with graphite monochromated Mo Kα radiation (*λ* = 0.71073 Å). Data reduction was carried out using the program Bruker SAINT.^47^ An empirical absorption correction SADABS^48^ was applied. The structures of the title compounds were solved by direct method and refined by the full-matrix-least-squares technique on *F*^2^ with anisotropic thermal parameters to describe the thermal motions of all non-hydrogen atoms using the programs SHELXS97 and SHELXL97,^49^ respectively. All calculations were carried out using PLATON^50^ and WinGX system Ver-1.64.^51^ All hydrogen atoms were located from difference Fourier map and refined isotropically. The CIFs have been deposited with CCDC no. 1857442–1857444 for compounds 2–4, respectively. A summary of crystal data and relevant refinement parameters are given in Table 1.

**Table 1:** Crystal data and structure refinement parameters for 2–4^a^

Structure	2	3	4
Empirical formula	C_14_H_16_ClN_5_O_8_	C_14_H_12_ClN_5_O_2_	C_14_H_16_BF_4_N_5_O_4_
Formula weight	417.77	317.74	405.13
Temperature (K)	293(2)	293(2)	293(2)
Wavelength (Å)	0.71073	0.71073	0.71073
Crystal system	Monoclinic	Monoclinic	Monoclinic
Space group	*P*2_1_/*c*	*C*2/*c*	*P*2_1_/*c*
*a*, *b*, *c* (Å)	12.646(5), 12.620(5), 11.356(5)	10.873(5), 17.015(5), 15.789(5)	12.6386(3), 12.6389(4), 11.2764(3)
*α*, *β*, *γ* (°)	90.000(5), 104.910(5), 90.000(5)	90.000(5), 93.762(5), 90.000(5)	90.00, 104.9830(10), 90.00
Volume (Å^3^)	1751.3(12)	2914.7(18)	1740.03(8)
*Z*/density (calc.) (mg m^−3^)	4/1.584	8/1.448	4/1.546
Absorption coefficient (mm^−1^)	0.276	0.277	0.141
*F*(000)	864	1312	832
Crystal size (mm^3^)	0.08 × 0.12 × 0.17	0.07 × 0.09 × 0.18	0.04 × 0.09 × 0.18
*θ* range for data collection	2.32–24.77	2.39–25.00	2.32–25.00
Limiting indices	−14 ≤ *h* ≤ 14	−12 ≤ *h* ≤ 12	−15 ≤ *h* ≤ 15
	−14 ≤ *k* ≤ 14	−20 ≤ *k* ≤ 20	−15 ≤ *k* ≤ 15
	−13 ≤ *l* ≤ 13	−18 ≤ *l* ≤ 18	−13 ≤ *l* ≤ 13
Reflections collected/unique	23 893/2940 [*R*(int) = 0.0321]	18 378/2503 [*R*(int) = 0.0642]	22 670/2998 [*R*(int) = 0.0239]
Completeness to *θ* (%)	98%	98%	98.2%
Absorption correction	Multi-scan	Multi-scan	Multi-scan
Max. and min. transmission	0.98 and 0.96	0.98 and 0.97	0.995 and 0.985
Refinement method	Full-matrix least-squares on *F*^2^	Full-matrix least-squares on *F*^2^	Full-matrix least-squares on *F*^2^
Data/parameters	2940/281	2503/210	2998/280
Goodness-of-fit on *F*^2^	1.035	1.038	1.057
Final *R* indices [*I* > 2*σ*(*I*)]	*R*_1_ = 0.0406, w*R*_2_ = 0.1098	*R*_1_ = 0.0384, w*R*_2_ = 0.1032	*R*_1_ = 0.0428, w*R*_2_ = 0.1130
*R* indices (all data)	*R*_1_ = 0.0467, w*R*_2_ = 0.1148	*R*_1_ = 0.0442, w*R*_2_ = 0.1107	*R*_1_ = 0.0491, w*R*_2_ = 0.1185
Largest diff. peak and hole (e Å^−3^)	0.443 and −0.320	0.457 and −0.350	0.401 and −0.289

a *R*_1_ = ∑||*F*_o_| − |*F*_c_||/∑|*F*_o_|, w*R*_2_ = [∑{(*F*_o_^2^ − *F*_c_^2^)^2^}/∑{w(*F*_o_^2^)^2^}]^1/2^, w = 1/{*σ*^2^(*F*_o_^2^) + (*aP*)^2^ + *bP*}, where *a* = 0.0627 and *b* = 0.8450 for 2; *a* = 0.0663 and *b* = 1.4042 for 3; *a* = 0.0605 and *b* = 0.7933 for 4. *P* = (*F*_o_^2^ + 2*F*_c_^2^)/3 for all the structures.

### 2.6. Device fabrication

In this study, multiple metal–semiconductor (MS) junction devices were fabricated in ITO/compound (1–4)/Al sandwich structure to perform the electrical study. In this regard, well dispersion of the synthesized compounds (1–4) were made in *N*,*N*-dimethyl formamide (DMF) by mixing and sonicated the right proportion (30 mg ml^−1^) of compounds in separate vials. This newly prepared stable dispersion of compounds were deposited on the top of the ITO coated glass substrate by spun firstly at 600 rpm for 5 min and thereafter at 1000 rpm for 5 min with the help of SCU 2700 spin coating unit. Afterward, all the as-deposited thin films were dried in a vacuum oven (at a base pressure of 5 × 10^−3^ torr) at 80 °C for several minutes to evaporate the solvent part fully. The thicknesses of the developed films were measured by surface profiler as ∼1 μm. The aluminum electrodes were deposited under base pressure (10^−6^ torr) by maintaining the effective area as 7.065 × 10^−2^ cm^−2^ with shadow mask in the Vacuum Coating Unit 12A4D of HINDHIVAC. For electrical characterization of the devices, the current–voltage (*I*–*V*) characteristic was measured under both dark and AM 1.5G radiation condition and recorded with the help of a Keithley 2635B sourcemeter by two-probe technique. All the preparation and measurements were performed at room temperature and under ambient conditions.

### 2.7. Computational details

The *P*2_1_/*c* primitive monoclinic crystal structures of 2 and 4, and *C*2/*c* primitive monoclinic crystal structure of 3 were optimized with the density functional theory method using the CASTEP program code of Accelrys, Inc.^52^ They were relaxed with the experimental unit cell parameters fixed. The calculations were performed within the Generalized-Gradient approximation (GGA) and the Perdew–Burke–Ernzerhof (PBE) formulation for the exchange–correlation functional.^53,54^ Ultrasoft pseudopotentials^55^ were used in this work with the relativistic treatment of the Koelling–Harmon.^56^ A plane-wave basis set with 300 eV cut-off was applied. The k-mesh points over the Brillouin zone were generated with parameters 1 × 1 × 1 the Monkhorst–Pack-scheme. The energy tolerance for self-consistent field (SCF) convergence was 2 × 10^−6^ eV per atom for all calculations. The long-range dispersion correction has been included in the calculations with the Tkatchenko–Scheffler scheme.^57^ Band structures were calculated along the *k*-vector of the first Brillouin zone of the crystal and Partial density of states (PDOS) was plotted concerning the Fermi level with a 2 × 2 × 2 grid for compounds 2–4. The optical properties including dielectric function and optical conductivity of the crystal are calculated. Optical properties are calculated for plane polarized light with the specified polarization directions: (100) (010) (001). The smearing of 0.2 eV was employed.

### 2.8. Molecular docking studies

The selected B-DNA duplex sequences containing 10 base pairs each with a central core consisting of four specific bases from the 5′-end in each DNA duplex were used for docking study. To understand the binding mode, one representative salt (2) was docked into four DNA decamer sequences.^58,59^ The binding site of DNA groove was determined by site finder and the compound was placed accordingly.

Docking calculations using MOE program was conducted using Alpha PMI as the placement methodology. The docked pose obtained thus was further refined using force field method available in the MOE program.

### 2.9. Absorbance spectral studies

Shimadzu Pharma Spec UV-1700 spectrophotometer connected with a PC equipped with a thermoelectrically controlled cell holder (model TCC 240A) under stirring at 25 ± 0.1 °C in quartz cells of 1.00 cm path length was used to measure absorbance.

### 2.10. Circular dichroic study

Circular dichroic measurement was performed by a JASCO: J-815 Spectropolarimeter controlled by a PC. A rectangular quartz cell having 1.00 cm path length was used for all CD measurements at 25 ± 0.5 °C.

## 3. Results and discussion

### 3.1. TGA and PXRD analyses

PXRD has been carried out at room temperature with the powdered sample of the compounds 2–4. The major peaks of the PXRD pattern of the synthesized compounds (2–4) match well with the simulated pattern generated from the single crystal data, representing phase purity of the bulk (Fig. S6†). Both the compounds 2 and 4 contain two water molecules in their asymmetric unit. To study the PXRD pattern of the compounds without crystal water TGA of the compounds 2 and 4 was performed in the temperature range of 30 °C to 850 °C under N_2_ atmosphere. The TGA plots indicate a weight loss of 7.76% (calc. 8.63%) for the compound 2 in the temperature range 120–130 °C corresponds to the loss two lattice water molecules whereas the compound 4 loses two lattice water molecules in the range of 130–135 °C (expt. 7.62%; calcd, 8.88%) (Fig. S7†). PXRD patterns of the dehydrated 2 and 4 are shown in Fig. S8† that indicates the crystalline nature is sufficiently retained even after dehydration.

### 3.2. Crystallographic analyses

**Fig. 2:**
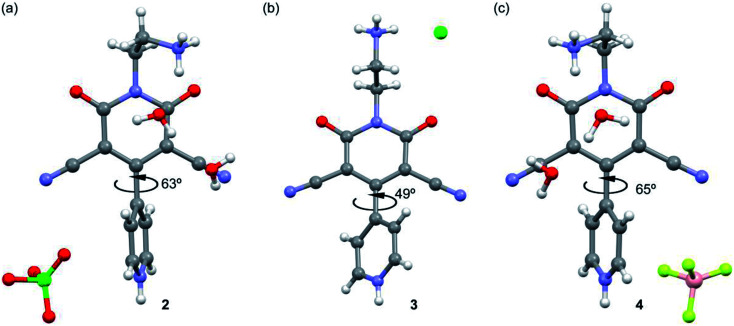
X-ray structures of compounds 2 (a), 3 (b) and 4 (c) with indication of the bis-arene torsion angle.

The asymmetric units of the title compounds of 2–4 consist of 4-(4-pyridinyl)-2-pyridone moiety with one protonated pyridine ring along with different anions and solvent water molecules (Fig. 2). We show in Fig. 2 the X-ray structure of compounds 2–4 and a detailed analysis of the effect of the different anions upon the supramolecular assemblies that govern the crystal packing is given in the ESI and in Tables S1–S3.† The main difference between the structures of the three compounds is the bis-arene torsion angle and the orientation of the ethylammonium group that strongly influences the solid state architecture of the compounds as detailed in Fig. S1–S5.†

### 3.3. Optical characterization

**Fig. 3:**
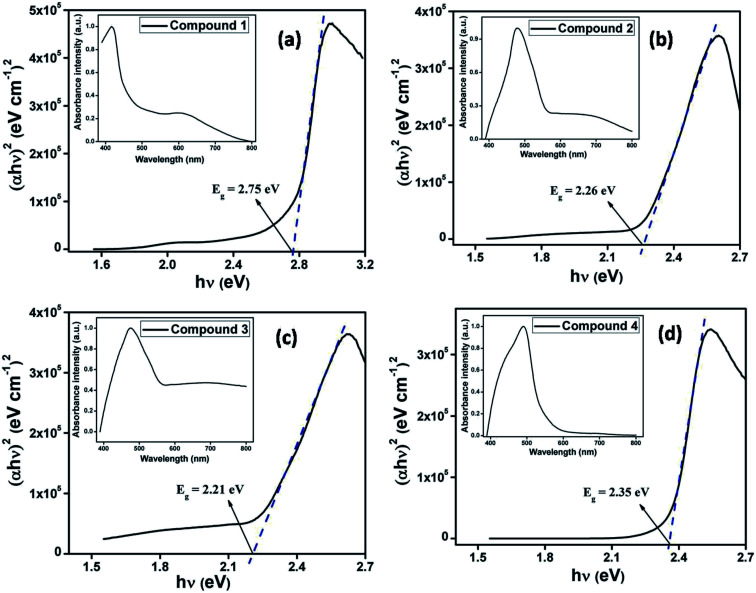
UV-vis absorption spectra (inset) and Tauc's plots for compounds 1–4.

The optical characterization of each compounds (1–4) have been performed based on UV-vis spectrum. As the synthesized compounds produce stable dispersion in DMF, thin films on normal glass substrate were prepared for solid-state UV-vis spectroscopy. The optical spectra of compounds 1–4 (inset Fig. 3) have been measured in the range 400–800 nm. The optical band gap for each of the films has been estimated from UV-vis spectrum using Tauc's equation (eqn (1)).^60^

1 *αhν* = *A*(*hν* − *E*_g_)^*n*^

where, *α*, *E*_g_, *h* and *ν* stands for absorption coefficient, band gap, Planck's constant and frequency of light. The exponent ‘*n*’ is the electron transition processes dependent constant. ‘*A*’ is a constant which is considered as 1 for ideal case. To calculate the direct optical band gap, the value of the exponent ‘*n*’ in the above equation has been considered as *n* = ½.^60^ By extrapolating the linear region of the plot (*αhν*)^2^*vs. hν* [Fig. 3(a–d)] to *α* = 0 absorption, the values of direct optical band gap (*E*_g_) of the compounds have been calculated as 2.75 eV, 2.26 eV, 2.21 eV and 2.35 eV for synthesized compounds 1–4, respectively.

### 3.4. Electrical characterization

The suitable optical band gap suggests that our synthesized compounds are semiconducting material. Hence, we have fabricated metal (Al)–semiconductor (synthesized compounds) (MS) junction thin film device and studied its electrical parameters by analyzing the charge transport behaviour. To analyze the electrical properties, current–voltage (*I*–*V*) measurements of compound 1–4 based multiple devices have been recorded with a Keithley 2635B sourcemeter under dark and AM 1.5G condition at corresponding applied bias voltage sequentially within the limit ±2 V.

**Fig. 4:**
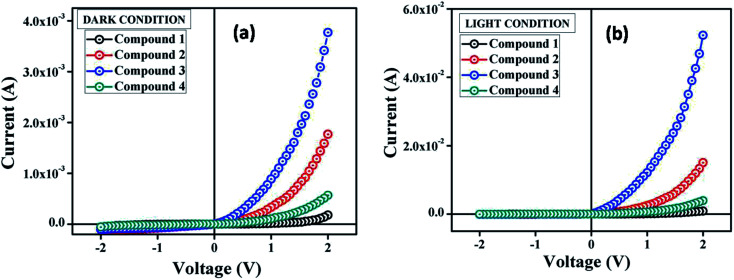
*I*–*V* characteristics curve for ITO/compound (1–4)/Al structured thin film devices under (a) dark, and (b) photo illumination condition.

The *I*–*V* characteristics of synthesized compound 1–4 based devices have been recorded under dark and irradiation condition and presented in Fig. 4. Under dark condition, the electrical conductivity has been estimated as 1.48 × 10^−6^, 4.11 × 10^−5^, 1.19 × 10^−4^ and 1.31 × 10^−5^ (in S m^−1^) for the compound 1–4 based devices respectively, typical of a semiconductor. However, after exposed under photoirradiation, the conductivity has been estimated as 1.27 × 10^−5^, 3.16 × 10^−4^, 1.68 × 10^−3^ and 8.33 × 10^−5^ (S m^−1^) for the compound 1–4 based devices, respectively. It is clear that the conductivity of all the devices improves significantly under irradiation conditions from the non-irradiated conditions. Moreover, the representative *I*–*V* characteristics of the Al/compounds interface under both dark and a photo illumination condition represents the nonlinear rectifying behaviour, similar to the Schottky barrier diode (SBD). The rectification ratio (*I*_on_/*I*_off_) of the SBDs under dark condition at ±2 V has been obtained as 2.32, 16.26, 37.03 and 9.80 for the compounds 1–4 based devices, respectively. Whereas, under photo illumination condition the same has been evaluated as 11.62, 36.54, 77.92 and 28.11 for the compound 1–4 based devices, respectively. The larger current from the characteristics curve under irradiation condition demonstrates the photoresponsivity of the devices, which has been found to be 5.00, 8.51, 13.87 and 6.99 for compound 1–4 based SBDs, respectively.

The *I*–*V* characteristic of the compound 1–4 based SBDs have been further analyzed by thermionic emission theory and Cheung's method was employed to extract important diode parameters.^60^ In this regard, we have analysed *I*–*V* curves quantitatively by considering the following standard equations:^60,61^2
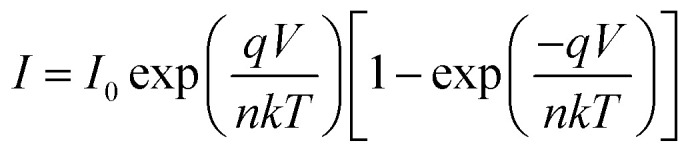
3
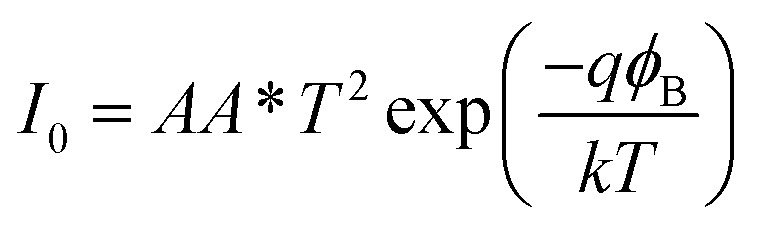
where *I*_0_, *k*, *T*, *V*, *A*, *η* and A* stands for saturation current, electronic charge, Boltzmann constant, temperature in kelvin, forward bias voltage, effective diode area, ideality factor and effective Richardson constant, respectively. The effective diode area has been estimated as 7.065 × 10^−2^ cm^2^ and the effective Richardson constant has been considered as 32 A K^−2^ cm^−2^ for all the devices.

The main problem for Schottky diode is their fairly high reverse leakage current compared to normal p–n junction diodes.^62^ At −2 V the leakage current for our synthesized compounds based Schottky diodes has been measured in the order of 10^−5^ A (Fig. 4). Generally, reverse leakage current in metal/semiconductor SBD arises due to the thermionic emission over the barrier^63^ and it is normally attributed to saturation of reverse bias current (eqn (3)). In the reverse bias regime, the leakage current observed at the Schottky interface can be explained using the thermionic-field emission (TFE) model,^64^ particularly for wide-band-gap materials.^65,66^

We have also determined the series resistance, ideality factor and barrier potential height by using eqn (4)–(6), which has been extracted from Cheung's idea,^67,68^4
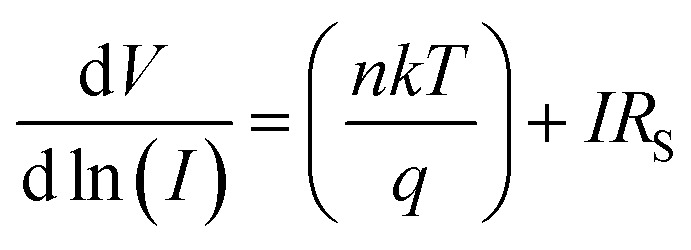
5
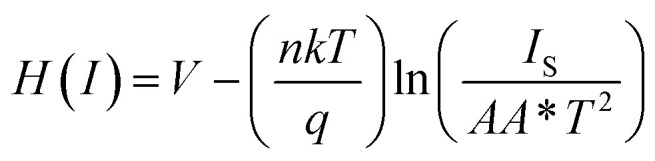

6 *H*(*I*) = *IR*_S_ + *ηϕ*_B_

**Fig. 5:**
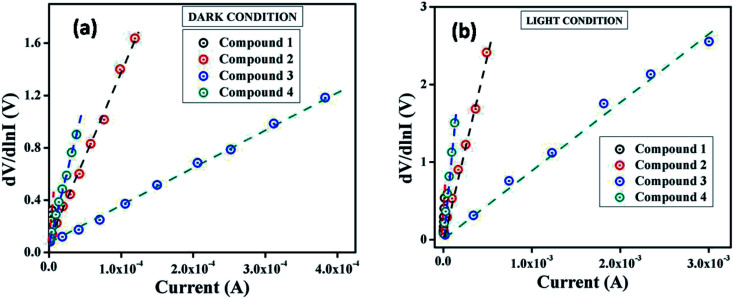
d*V*/d ln *I vs. I* curve for the compound 1–4 based thin film devices under (a) dark, and (b) photoillumination condition.

From the intercept of d*V*/d ln *I vs. I* plot (Fig. 5) the ideality factor (*η*) for all devices under both conditions has been determined whereas the slope of this plot represents the series resistance (*R*_S_) of the devices. The obtained value of ideality factors for all the devices both under dark and irradiation conditions are listed below in Table 2. The values of ideality factor (*η*) have been estimated as 3.41, 2.82, 2.43 and 3.17 under dark condition for compound 1–4 based SBDs, respectively. Under photo illumination condition the same has been estimated as 3.24, 2.20, 1.56 and 2.89 for compound 1–4 based SBDs, respectively. The obtained values of ideality factors of all the devices under both the conditions represent a deviation from its ideal value (∼1). This may be due to the presence of heterogeneities of Schottky barrier height and existence of interface states and series resistance at the junction.^69,70^ However, under irradiation condition the values of ideality factor for all the compounds based SBDs approaches more ideal (closer to 1), which is a significant observation. In general, it depicts the fewer number of recombination of interfacial charge carriers and generation of better homogeneity at the barrier of Schottky junctions.^60^ Furthermore, under both conditions the values of ideality factors of compound 3 based SBD approaches more ideal rather than the rest of the compound based devices. From this, it may be concluded that our synthesized compound 3 possesses less carrier recombination at the junction *i.e.* better barrier homogeneity even under photo irradiation condition than all other synthesized compounds based devices.

**Table 2:** Schottky device parameters of compound 1–4 based SBDs

Device	Condition	On/off	Conductivity (S m^−1^)	Photosensitivity	Ideality factor	Barrier potential height (eV)	*R*_S_ from d*V*/d ln *I* (kΩ)	*R*_S_ from *H* (kΩ)
1	Dark	2.32	1.48 × 10^−6^	5.00	3.41	0.43	59.06	55.03
	Light	11.62	1.27 × 10^−5^		3.24	0.42	44.11	40.79
2	Dark	16.26	4.11 × 10^−5^	8.51	2.82	0.38	13.05	11.57
	Light	36.55	3.16 × 10^−4^		2.20	0.35	4.81	3.06
3	Dark	37.03	1.19 × 10^−4^	13.87	2.43	0.35	2.93	1.01
	Light	77.92	1.68 × 10^−3^		1.56	0.31	0.99	0.86
4	Dark	9.80	1.31 × 10^−5^	6.99	3.17	0.41	21.44	19.64
	Light	28.00	8.33 × 10^−5^		2.89	0.39	11.18	10.49

**Fig. 6:**
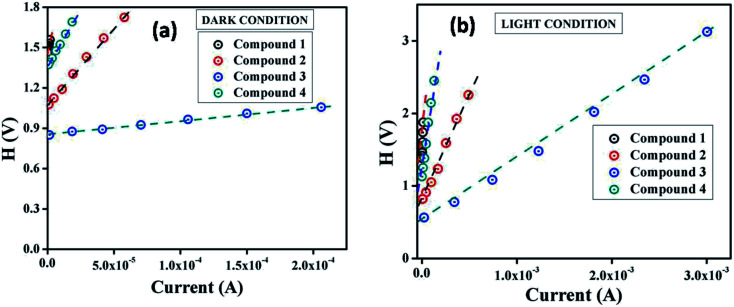
*H vs. I* curves for the compound 1–4 based thin film devices under (a) dark, and (b) photo illumination condition.

From the intercept of *H*(*I*) *vs. I* plot (Fig. 6) and using the calculated ideality factor (*η*) values, the value of barrier potential height (*ϕ*_B_) has been determined [eqn (6)].

The significant observation is that the barrier potential height of all the devices is reduced under irradiation condition. The generation of photo induced charge carriers and their accumulation near the conduction band may be the main reason for this diminution. The series resistance (*R*_S_) of the devices can also be determined from the slope of this plot. The measured barrier potential height (*ϕ*_B_), ideality factor (*η*) and series resistance (*R*_S_) under dark and illumination condition for the metal (Al)–semiconductor (synthesized compounds) (MS) junctions are listed in Table 2. The series resistance obtained from both processes show good consistency. The obtained series resistance is found to decrease upon light illumination (Table 2), which signifies its applicability in the field of optoelectronics devices.

Table S4† compares conductivity data of our synthesized compounds 1–4 with some of the reported organic and metal–organic compounds (suggestions of a referee) that shows compound 3 is a potentially promising candidate for organic semiconducting materials.

In the simple case of a biphenyl where a molecule with two phenyl rings linked by a single C–C bond, conductance is expected to change with the relative twist angle between the two rings.^27^ The planar conformation is expected to have the highest conductivity.^71^ Compounds 1–4 are closely related to above said biphenyl systems. From the crystal structures of the salts (2–4), it is established that the two groups (amino and substituted 4-(4-pyridinyl)-2-pyridone) attached with ethyl moiety of compound 3 are in anti conformation, whereas they are in gauche conformation in 2 and 4. The aromatic rings of these kinds of systems deviate from planarity due to steric congestion and this deviation is greater for *gauche* conformation. Torsion angles of corresponding compounds increase with increasing deviation from planarity. Consequently, extent of π conjugation between the two aromatic rings decreases and energy gap (band gap) between valance band and conduction band increases. In 3, torsion angle (deviation from planarity) is minimum; electron movement is this expected to be greater. This result is reflected in UV-vis spectra where transition starts at higher wavelength (∼561 nm) for compound 3 and it is associated with lower optical band gap (2.21 eV). This trend is followed in 2 (where, torsion angle is greater than 3, transition starts at wavelength ∼548 nm) and in 4 (where, torsion angle is even greater than that in 2, transition starts at wavelength ∼530 nm). Thus change in twist (torsion) angles in 2–4, the UV-vis spectrum, band gaps and their semiconducting properties were also found to be different.

**Fig. 7:**
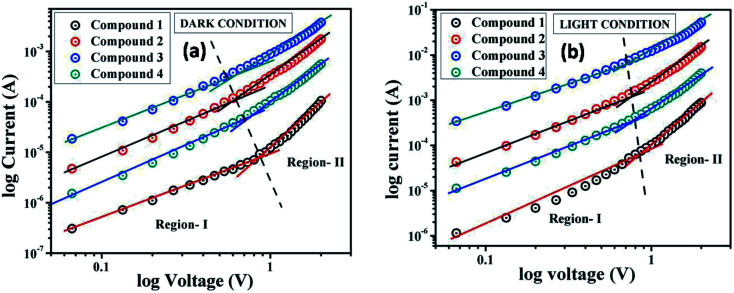
log *I vs.* log *V* curves for the compound 1–4 based thin film devices under (a) dark, and (b) irradiation condition.

For a better understanding of the charge transport phenomena at MS junction, it requires an analysis of the *I*–*V* curves in details. The characteristic *I*–*V* curves under both conditions in the logarithmic scale reveals that it can be differentiated in two slopes (Fig. 7), which has been marked as region-I and II.

In the first region, when the value of slope is ∼1, current follows the relation *I* ∝ *V*, which refers to the ohmic regime. In the second region, the value of slope is about 2, where current is proportional to *V*^2^ (Fig. 7). This is the characteristic of a trap free space charge limited current (SCLC) regime.^60,72^ If the injected carriers are more than the background carriers, the injected carriers spread and generate a space charge field. The currents are controlled by this space charge field and are known as SCLC.^60,72^ To estimate the device performance here we have been adopted this SCLC theory.

**Fig. 8:**
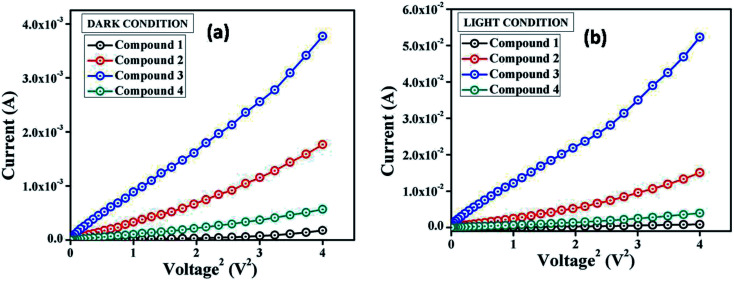
*I vs. V* ^2^ curves for the compound 1–4 based thin film devices under (a) dark, and (b) irradiation condition.

Following this model, the effective carrier mobility was estimated from higher voltage region of *I vs. V*^2^ plot (Fig. 8) by Mott–Gurney equation:^60,68,72^7
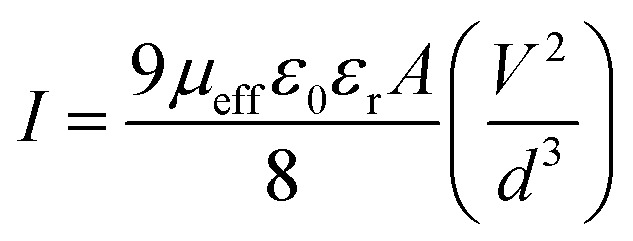
where, *I* is the current, *ε*_0_ is the permittivity of free space, *ε*_r_ is the relative dielectric constant of the synthesized material, *μ*_eff_ is the effective dielectric constant.

**Fig. 9:**
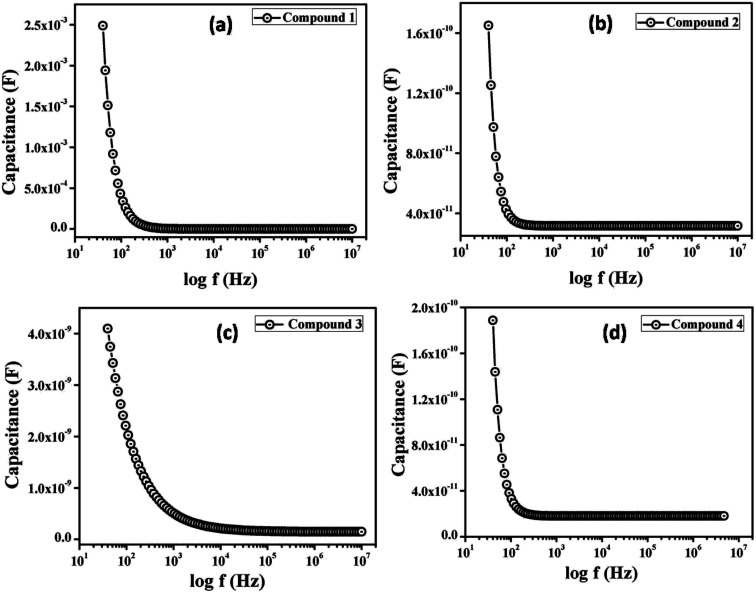
Capacitance *vs.* frequency graph for determination of dielectric constant.

To measure the relative dielectric constant, we have drawn the capacitance against frequency of synthesized material in film format at constant bias potential (Fig. 9).

From the saturated values of capacitance at the higher frequency regime (Fig. 9) the dielectric permittivity of the compounds has been calculated using following equation:^60^8
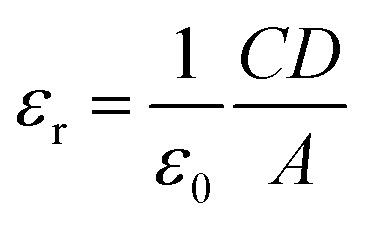
where, *C* is the capacitance (at saturation), *D* is the thickness of the film which has been considered as ∼1 μm and *A* is the effective area. Using the above formula the relative dielectric constant of the material has been estimated as 1.73 × 10^−1^, 5.05 × 10^−1^, 8.52 × 10^−1^ and 2.88 × 10^−1^ for compounds 1–4 respectively. Transit time (*τ*) and diffusion length (*L*_D_) are few more key parameters have also been estimated to analyze charge transport across the junction. For this purpose *τ* has been evaluated from eqn (9), by using the slope of SCLC region (region II) in logarithmic representation of forward *I*–*V* curve, shown in Fig. 7.^60^9
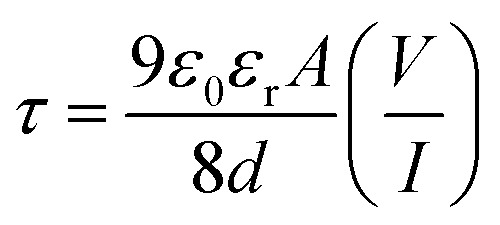
10
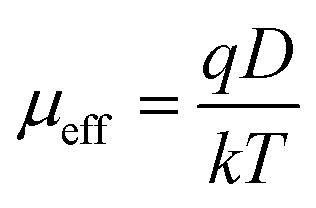
11
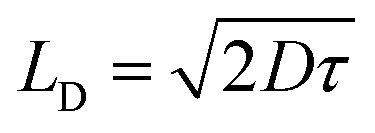
where, *D* is the diffusion coefficient and has been determined using Einstein–Smoluchowski equation (eqn. (10)).^60^ When a metal semiconductor junction is formed, diffusion length (*L*_D_) of charge carriers plays an influential role in device performance and has further been extracted from the eqn (11). All the parameters estimated in the SCLC region demonstrate that the charge transport properties of the material improve after light soaking (Table 3). The higher mobility implied higher transport rate under irradiation, while the number of charge carriers also increased under the same condition. The increased diffusion length under illumination reveals that the charge carriers got to travel more length before being recombined, which led to the eventual increase in current displayed by the device under light. The diode parameters of the compound 3 based SBD indicate that it is of superior performance as compared to rest of the synthesized compounds. Compound 3 based SBD also demonstrates much enhanced charge transfer kinetics after light soaking. So, these kinds of materials can pave the way for a very promising future in device application.

**Table 3:** Charge conducting parameters of the compounds 1–4 based thin film devices

Device	Condition	*ε*_r_	*μ*_eff_ (m^2^ V^−1^ s^−1^)	*τ* (sec)	*μ*_eff_ *τ*	*D*	*L*_D_ (m)
1	Dark	1.73 × 10^−1^	3.82 × 10^−11^	7.78 × 10^−3^	2.97 × 10^−13^	9.55 × 10^−13^	1.22 × 10^−7^
	Light		1.81 × 10^−10^	1.71 × 10^−3^	3.06 × 10^−13^	4.50 × 10^−12^	1.24 × 10^−7^
2	Dark	5.05 × 10^−1^	1.05 × 10^−10^	3.83 × 10^−3^	4.01 × 10^−13^	2.62 × 10^−12^	1.42 × 10^−7^
	Light		9.42 × 10^−10^	4.45 × 10^−4^	4.19 × 10^−13^	2.35 × 10^−11^	1.45 × 10^−7^
3	Dark	8.52 × 10^−1^	1.26 × 10^−10^	2.35 × 10^−3^	4.22 × 10^−13^	3.15 × 10^−12^	1.22 × 10^−7^
	Light		17.22 × 10^−10^	2.73 × 10^−4^	4.70 × 10^−13^	4.31 × 10^−11^	1.53 × 10^−7^
4	Dark	2.88 × 10^−1^	5.96 × 10^−11^	5.49 × 10^−3^	3.27 × 10^−13^	1.49 × 10^−12^	1.28 × 10^−7^
	Light		4.39 × 10^−10^	7.74 × 10^−4^	3.39 × 10^−13^	1.09 × 10^−11^	1.30 × 10^−7^

### 3.5. Theoretical rationalization

**Fig. 10:**
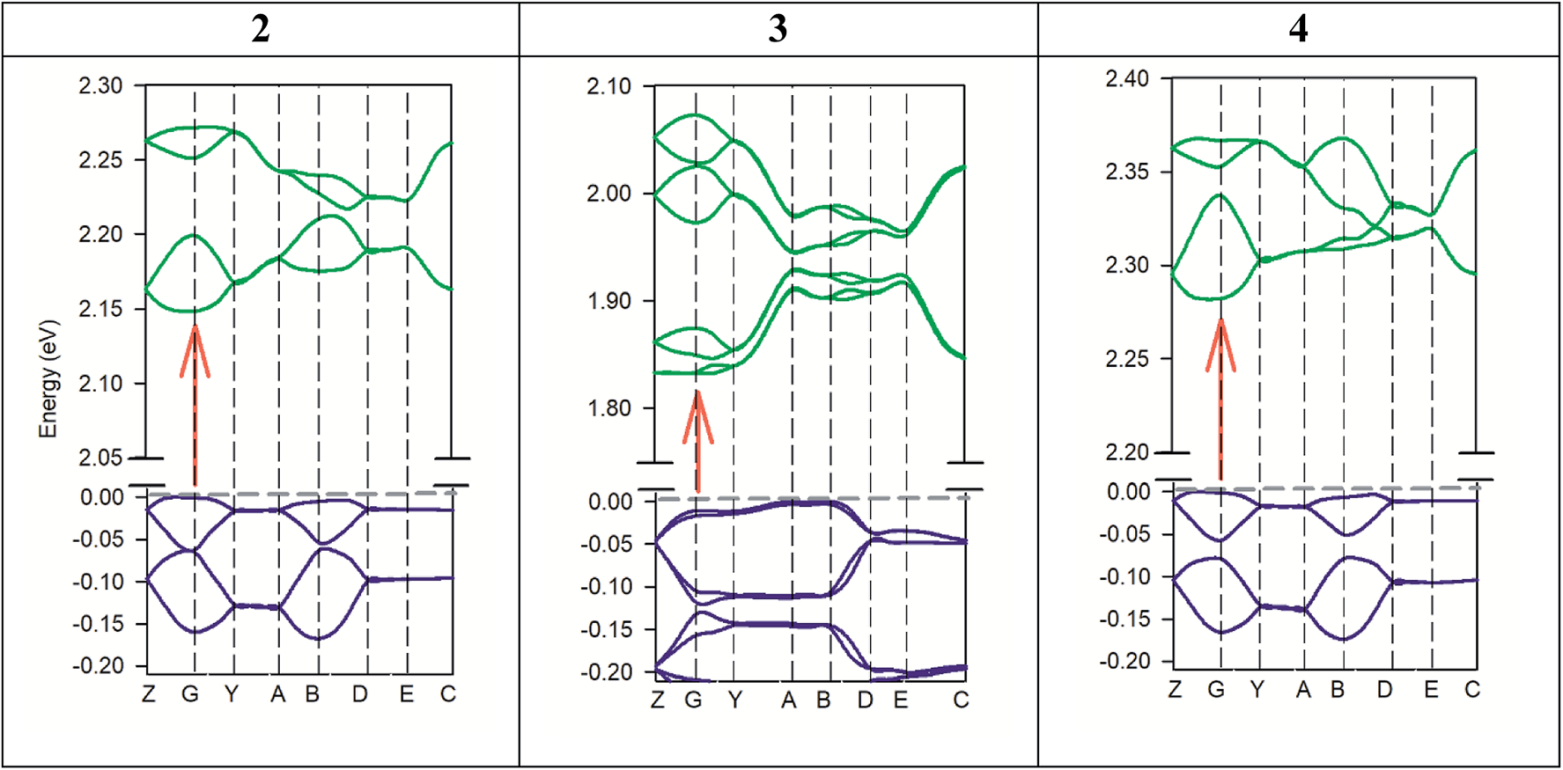
Electronic band structures of the ground state of 2, 3, and 4 crystals. Points of high symmetry in the first Brillouin zone are labeled as follows: Z = (0, 0, 0.5), G = (0, 0, 0), Y = (0, 0.5, 0), A = (−0.5, 0.5, 0), B = (−0.5, 0, 0), D = (−0.5, 0, 0.5), E = (−0.5, 0.5, 0.5), C = (0, 0.5, 0.5).

The experimental band gaps measured for the different crystals are quite similar ranging from 2.21 to 2.35 eV. However, significant differences in their electrical properties have been found both in the dark and when the material is illuminated with visible light (Table 2). Theoretical study is focused to rationalize this behaviour that should be related with the influence of the anion in the solid-state structure (Fig. S1–S5† and 2). The theoretical methodology used herein allows us to compute the properties of the different materials. However, we must keep in mind that other external effects can affect the experiments such as metal–semiconductor interfaces, semiconductor thickness, excitons, defects, and impurities, which are beyond this procedure. We have modelled the three different salts [perchlorate (2), chloride (3) and tetrafluoroborate (4)] using Density Functional Theory (DFT) with the experimental crystal lattices as starting points for the optimization of the atomic positions. Afterward, crystal structure analysis has been carried out by using the standard band theory (SBT) and the partial density of states (PDOS) calculation. For plotting the Kohn–Sham electronic energy levels as a function of the reciprocal space vector *k*, we have chosen a path along the first Brillouin Zone (BZ) passing through a set of high symmetry points depicted in Fig. 10.

The results from these calculations validate that all these compounds are semiconductors (Fig. 10). 2, 3 and 4 compounds crystallize as *P*2_1_/*c*, *C*2/*c* and *P*2_1_/*c* monoclinic crystals with a theoretical band gap of 2.15 eV, 1.83 eV and 2.28 eV. As it is known, DFT tends to underestimate semiconductor band gap values.^73,74^ Although the band gap values are slightly underestimated (especially, compound 3), it is considered that the shape of the bands is correct. In agreement with experiment, it has been verified that all compounds are direct semiconductors with the direct band gap in the Γ point of BZ. Compound 3 is the one with the lowest band gap followed by compound 2 and 4, also in agreement with experimental results. This fact can be related to the more significant number of accessible energy levels for the promotion of electrons from valence levels to conduction levels.

**Fig. 11:**
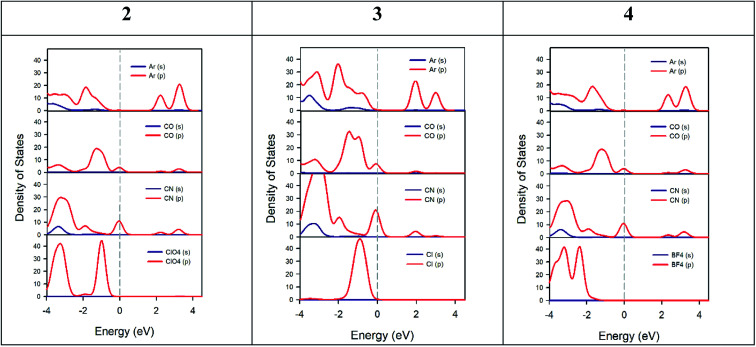
Calculated partial density of states of 2, 3 and 4 crystal cell. The left panel shows PDOS of aromatic atoms of AHPD (Ar), ketone group (CO), nitrile group (CN) and perchlorate anions (ClO_4_^−^) of 2 crystal cell. Middle panel shows of PDOS of aromatic atoms of AHPD (Ar), ketone group (CO), nitrile group (CN) and chloride anions (Cl^−^) of 3 crystal cell. The right panel shows the PDOS of aromatic atoms of AHPD (Ar), ketone group (CO), nitrile group (CN) and tetrafluoroborate anions (BF_4_^−^) of 4 crystal cell.

Fig. 11 shows the PDOS of the three crystal cells studied theoretically. This analysis helps us to understand which atoms or molecules are mainly involved in electrical conductivity. Likewise, the bonds between atoms in a crystal as well as electric transport phenomena are due to electrons from the outermost shell. For all crystals studied, the PDOS calculation shows that the top of valence bands is mainly dominated by p-orbitals of ketone and nitrile groups of the AHPD. Besides, the main contributors to the bottom of the conduction band are p-orbitals of the aromatic part of the AHPD. The counter anions used have levels only in the bottom of the valence band. Therefore, they do not participate in the conductivity properties of the materials directly. Their influence on the property is due to the changes that they provoke in the crystal packing due to their different size or orientation. This behaviour has been also found in other semiconductors like organic–inorganic metal halide perovskites with organic cations.^75,76^

To properly understand the electronic structure of the solid-state material, it is important to compute and analyze the optical properties. In each material, the frequency dependence of an incident photon can be studied by computing the dielectric function *ε*(*ω*) and the optical conductivity [*σ*(*ω*)]. The real part of *ε*(*ω*) gives information about the polarization degree of the material upon the application of an electric field and the imaginary part is a good indicator of the photon absorption. Moreover, *σ*(*ω*) is useful to analyze how the conductivity of the material changes upon illumination.^77^

**Fig. 12:**
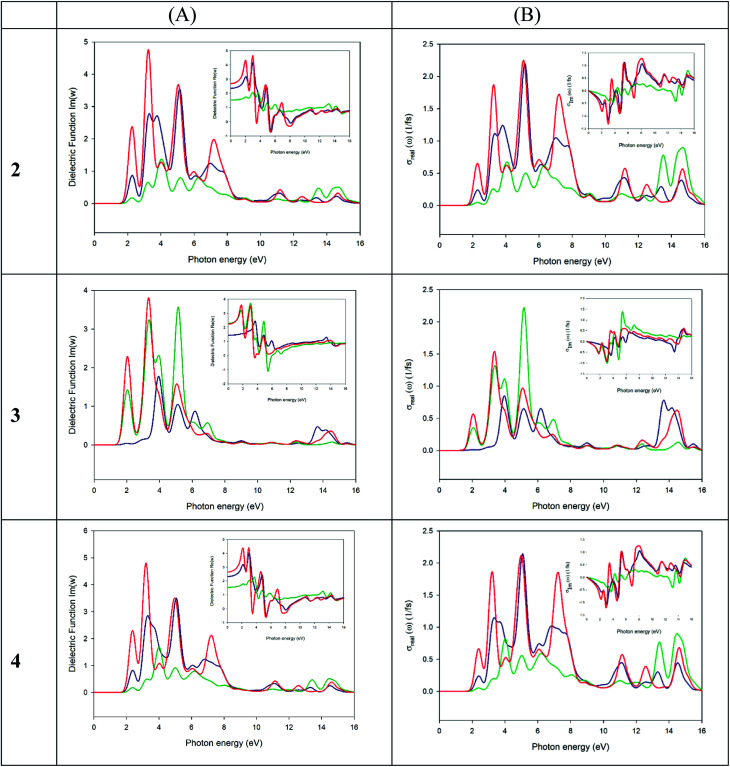
(A) A plot of real (small square) and imaginary (big square) parts of the dielectric function *versus* the photon energy of 2, 3 and 4 crystals. Blue, green and red lines specify the incident direction of polarized radiation in the ‘*x*’, ‘*y*’ and ‘*z*’ directions of the crystal, respectively. (B) A plot of real (big square) and imaginary (small square) parts of optical conductivity *versus* the photon energy of 2, 3 and 4 crystals. Blue, green and red lines specify the incident direction of polarized radiation in the ‘*x*’, ‘*y*’ and ‘*z*’ directions of the crystal, respectively.

We have used the computed band structure to measure the optical response (Fig. 12A). To do so, we have selected a photon energy range of 0–16 eV. The peaks can be assigned to electronic transitions from the top of the valence band to the low energy in the conduction band. Due to the anisotropy of the crystals we have decomposed the dielectric constant in three main directions where the light falls on the crystals (*ε*_*xx*_, *ε*_*yy*_, *ε*_*zz*_). In the crystal 2 (left panel, Fig. 12) the first maximum peaks of the three direction fall in the theoretical band gap ∼2.19 eV. As seen in the graph, the peaks in all directions of polarization of light are similar concerning to its width, presenting an absorption range between 1.6 to 8.5 eV, but not with respect to the intensity. Other maximum peaks can be seen in this range in a, 3.21, 5.03 and 7.09 eV. The 3 crystal has two preferred absorption directions within the crystals [*ε*_*yy*_ (green line) and *ε*_*zz*_ (red line)] starting at lower energy levels than 2, with the main absorption range between 1.32–7.35 eV and with maximums of 1.98, 3.29 and 5.13 eV. Finally 4 is the crystal with the highest band gap of the three studied, its preferred adsorption ranges between 1.76 and 8.36 eV, with maximums at 2.32, 3.16, 4.88 and 7.11 eV in the directions *ε*_*xx*_ (blue line) and *ε*_*zz*_ (red line) incident light. As can be seen, the behaviour of the optical conductivity (Fig. 12B) is similar to that presented by the dielectric constant. The calculation of optical properties shows that only some wavelength obtains an optical response. In our case, compound 3 has an absorption range of between 1.54–7.8 eV with maximums in 2.0, 3.4 and 5.2 eV, apparently more prone to the absorption of visible light than the crystals 2 and 4 with ranges between 1.8–8.5 eV and 1.9–8.5 eV respectively. These facts together with a lower band gap of the compound justify the higher electrical conductivity of the material and the higher electrical conductivity when the sample is illuminated.

### 3.6. DNA binding study

#### 3.6.1. Molecular docking studies

**Fig. 13:**
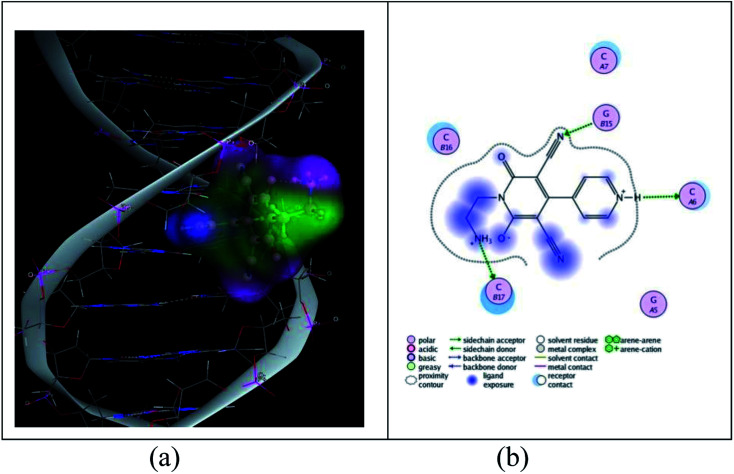
(a) Representative picture for the partial intercalation of cationic moiety into DNA base pairs. (b) 2-dimensional picture for the binding of cationic moiety with DNA.

DNA binding activity mainly depends on the structure of cationic moiety. Here the structures of all the cationic part are same (only the anion is different), so the activity is studied with compound 2 only. The cationic moiety of 2 partially intercalates into the base pair of DNA (Fig. 13) and it has been predicted by MOE program. The free energy of binding is in the range of −3.61 to −3.95 kcal mol^−1^. This value of UV-vis spectroscopic studies is in good agreement with the value obtained by docking study. Several hydrogen bonds formed between ‘N’ of CN, ‘O’, NH_2_ group of cationic moiety and DNA base during complex formations is the reason for extra stability of the complex.

The conformational free energy predicted here is nearly 0.8 kcal mol^−1^ (Table S5†) and it is also supported by circular dichroic spectral study.

#### 3.6.2. UV-visible study and evaluation of binding parameters

**Fig. 14:**
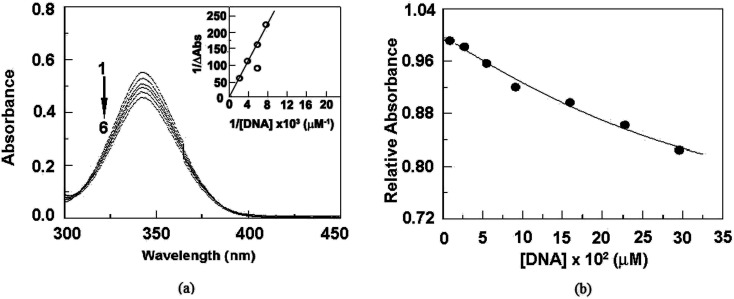
(a) Absorbance spectra of compound 2 35 μM in presence of 0, 196, 294, 417, 540 and 620 μM of DNA (plots 1 to 6) (inset: Benesi–Hildebrand analysis plot). (b) Relative absorbance change with increasing concentration of DNA.

The absorbance spectra of representative perchlorate salt are presented in Fig. 14 with increasing concentration of CT-DNA (Calf-Thymus DNA). Hypochromic effect was observed with increasing concentration of DNA. No isosbestic point was observed and this may be due to large molar extinction coefficient of organic compound than that of compound-DNA complex.^58^

Binding constants is determined to be order of 10^2^ (M^−1^) from the Benesi–Hildebrand plot through the binding of cationic moiety.^59^ Analysing the Benesi–Hildebrand plot the binding constant is found to be 1.48 × 10^2^ M^−1^. This result is also supported by molecular docking study and circular dichroic study.

#### 3.6.3. Circular dichroic study

**Fig. 15:**
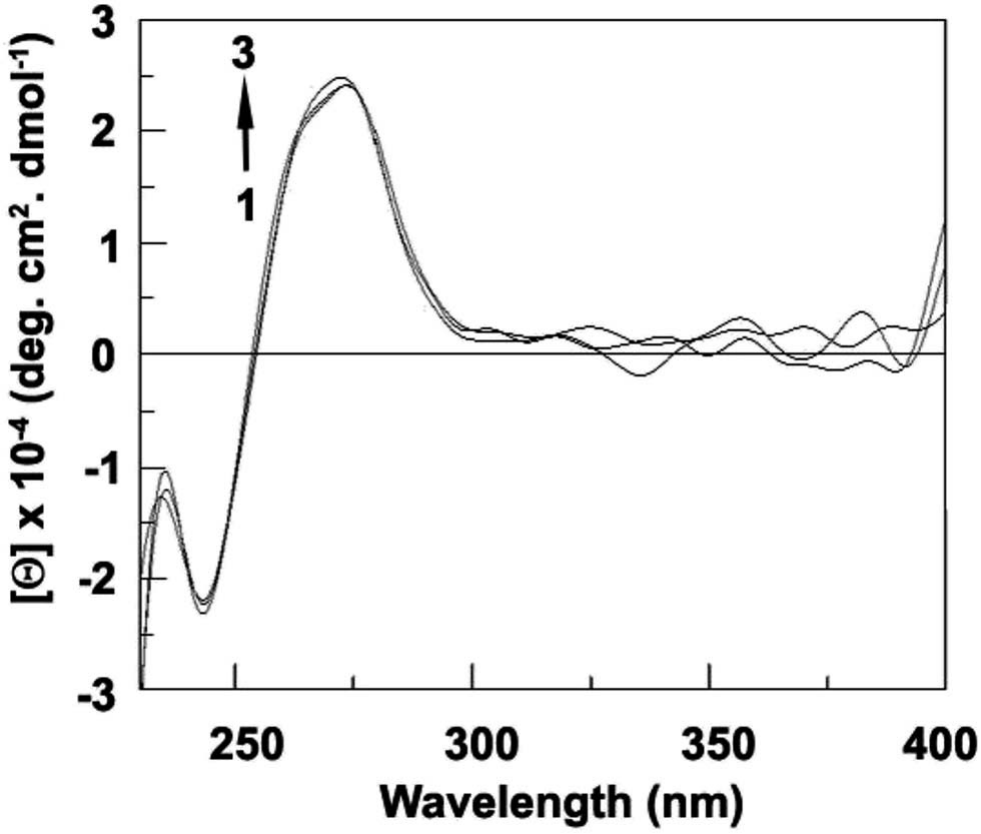
Intrinsic CD spectra of CT DNA (60 μM) in the presence of 0, 6, 12, 18, and 30 μM of compound 2.

Circular dichroic spectral study was performed to observe any conformational change of DNA during binding. DNA shows a positive band near 270 nm and negative band near 240 nm which clearly indicate B-form conformation of DNA. From our study we have observed both the positive and negative band change during binding. The change in positive band indicates that stacking between base pairs decreased whereas change in negative band indicates that conformation of DNA helix also changed (unwinding) (Fig. 15).

## 4. Concluding remarks

In conclusion, we have synthesized three salts from a mother organic compound and established their solid-state crystal structures. We have investigated the optical, dielectric and electrical properties of these salts and the mother compound. Optical band gaps of all four compounds were found to be in the semiconductor range. Salt formation substantially improved the electrical properties over the mother compound. The electrical conductivity of all the compounds in the Al/(1–4)/ITO configuration increases on illumination to visible light but the magnitude of enhancement is not same for the three salts which was also theoretically studied. It appears that these kinds of organic salts could open a new window in organic semiconducting industries as well as in the broad research field of optoelectronic devices. DNA binding study reveals that the compound is also biologically important as it has ability to bind DNA helix by partial intercalative manner.

## Conflicts of interest

There is no conflict to declare.

## Supplementary Material

RA-009-C9RA00666D-s001

RA-009-C9RA00666D-s002
