# Supplementary material for: Structures, photoresponse properties and DNA binding abilities of 4-(4-pyridinyl)-2-pyridone salts
Source: RSC Adv. 2019 Mar 27;9(17):9663–77. doi: 10.1039/c9ra00666d (PMC9062393; doi:10.1039/c9ra00666d)
Supplement: RA-009-C9RA00666D-s001 [file RA-009-C9RA00666D-s001.pdf]

Electronic Supplementary Material (ESI) for RSC Advances.
This journal is © The Royal Society of Chemistry 2019

Supporting Information

Structures, Photoresponse Properties and DNA Binding Abilities of 4-(4-Pyridinyl)-2-Pyridone Salts

Tripti Mandal,[a] Arka Dey,[b,c] Sudipta Pathak,[d] Md. Maidul Islam,[e] Saugata Konar,[f] Joaquín Ortega-Castro,[g] Saikat Kumar Seth,[*c] Partha Pratim Ray,[*c] Antonio Frontera,[*g] Subrata Mukhopadhyay[a]

[a]*Department of Chemistry, Jadavpur University, Jadabpur, Kolkata 700032, India*

[b]*Department of Condensed Matter Physics and Material Sciences, S. N. Bose National Centre for Basic Sciences, Block JD, Sec. III, Salt Lake, Kolkata 700106, India*

[c]*Department of Physics, Jadavpur University, Jadabpur, Kolkata 700032, India*

[d]*Department of Chemistry, Haldia Government College, Debhog, Purba Medinipur, West Bengal 721657, India*

[e]*Department of Chemistry, Aliah University, Action Area IIA/27, Kolkata 700156, India*

[f]*Department of Chemistry, Bhawanipur Education Society College, Bhowanipore, Kolkata 700020, India*

[g]*Departament de Química, Universitat de les Illes Balears, Crta. de Valldemossa km7.5, 07122 Palmade Mallorca (Baleares), Spain*

Structural description

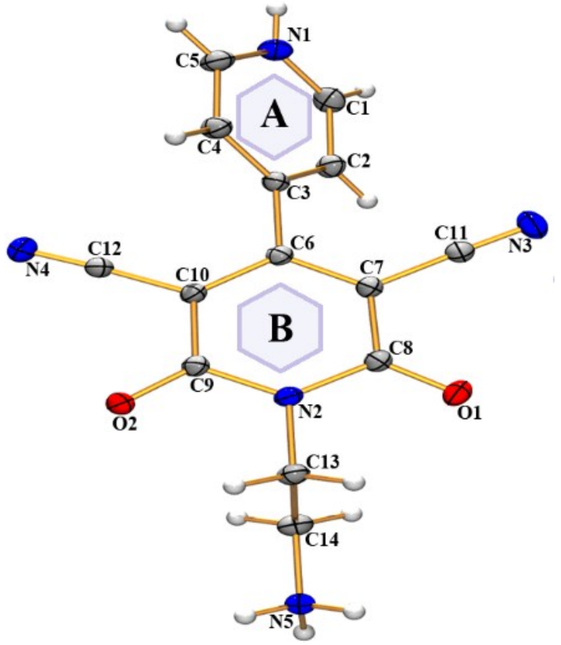

Fig. S1 ORTEP diagram and atom-numbering scheme of the cationic moiety of compounds **2**–**4**. Anions and solvent molecules are omitted for the sake of clarity. Thermal ellipsoids are drawn at 30% probability.

The solid-state structure of **2** includes a combination of N–H···N, N–H···O, O–H···O, C–H···N and C–H···O hydrogen bonds along with π···π stacking, lp···π and anion···π^+ interactions (Tables S1–S3). In the first substructure, the solvent water molecules play a crucial role in building supramolecular network structure. The water oxygen atom O(8) in the molecule at (x, y, z) acts as double donor to the oxygen atoms O(3) and O(5) in the molecule at (2–x, 1/2+y, 3/2–z) and (x, 1/2–y, 1/2+z) respectively; thus forming a $R_4^4(12)$ ring motif (M) (Fig. S2a). The separation distance between O(4) and O(6) atoms of two parent and partner perchlorate ions is 2.988 Å which interconnects the water-anion ring motifs and leads the network to propagate along a one-dimensional chain. Again, another solvent water molecule acts as double donor to the water oxygen atom O(8) of the ring motif and to the carbonyl oxygen atom O(1) of the

cationic moiety; thus interconnects the water anion cluster with the cationic moiety and leads the molecules to generate a two-dimensional supramolecular network in (101) plane (Fig. S2a). In another sub-structure, the protonated pyridine nitrogen atom N(1) at (x, y, z) acts as donor to the N(3) atom of the partner molecule at (2–x, –y, 1–z); thus forming a centrosymmetric $R_2^2(18)$ dimeric ring (N) which is centered at (1, 0, ½) (Fig. S2b). Due to the self-complementarity nature, the amine nitrogen atom N(5) at (x, y, z) interact with the nitrogen atom N(4) of the partner molecule at (x, 1/2–y, –1/2+z); thus interconnects the dimers and leading to the formation of a two-dimensional supramolecular network in (011) plane (Fig. S2b). In another sub-structure, the carbonyl oxygen atom O(2) in the molecule at (x, y, z) is oriented toward the π-cloud of the B-ring of the partner molecule at (1–x, –y, 1–z) with a distance of 3.380(2) Å, suggesting lp···π interaction.[1,2] The self-complementary nature leads the molecules to form a dimer through lp···π interaction (Fig. S2c). Moreover, the perchlorate oxygen atom O(3) is juxtaposed to the π-cloud of the protonated A-ring, displaying significant anion···π^+interaction[3,4] where the distance between the O(3) atom and the centroid of the A-ring is 3.609(3) Å. Thus, the cooperative nature of the noncovalent lp···π and anion···π^+ interactions increase the strength of the molecular assembly in **2** (Fig. S2c).

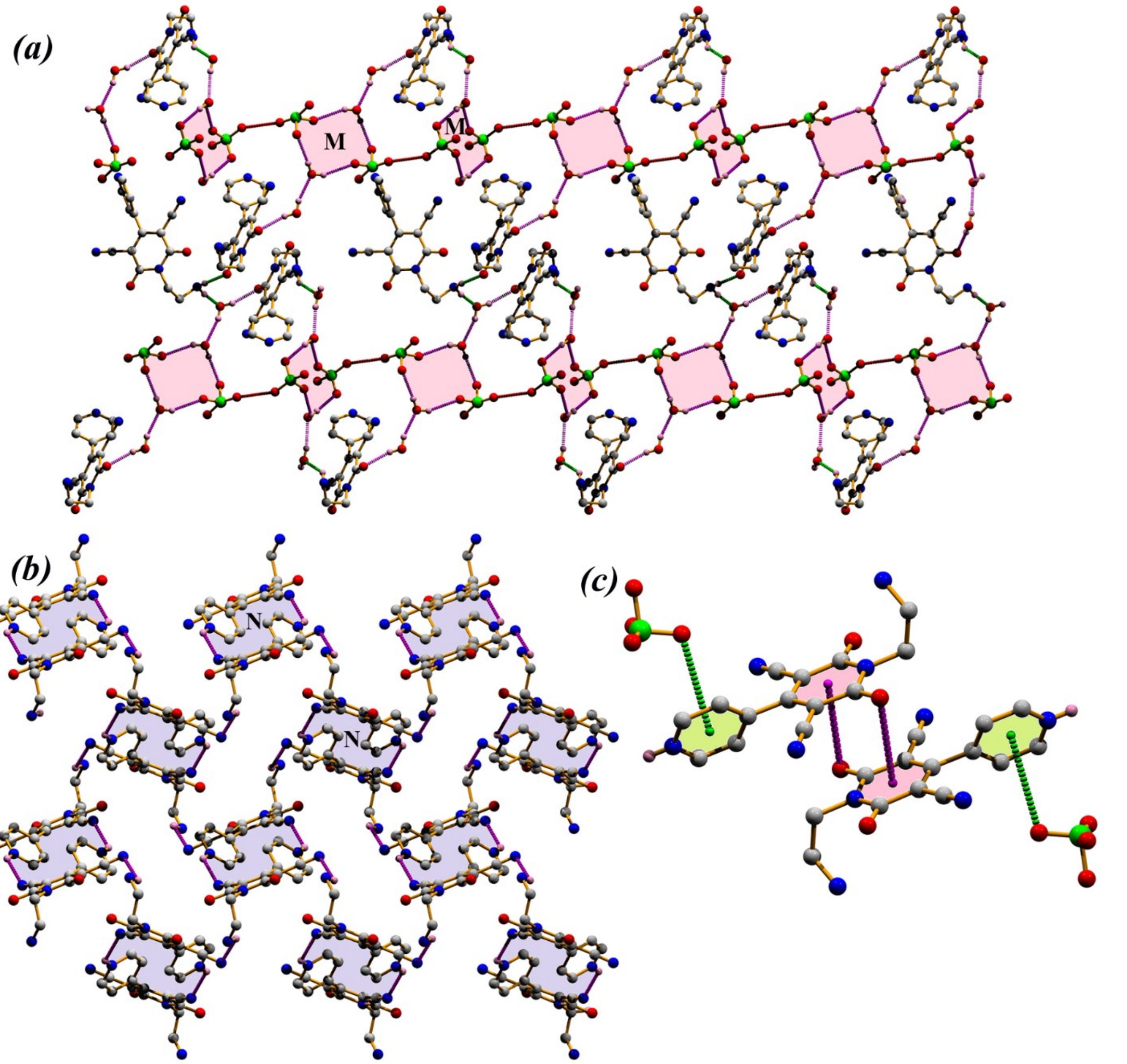

Fig. S2 (a) Formation of water-anion cluster and the two-dimensional network in (101) plane, (b) zero-dimensional dimeric unit acting as building block for the formation of two-dimensional supramolecular network, (c) cooperativity of lp···π and anion···π^+ interactions in **2**.

The crystal structure of **3** exhibits N–H···Cl, N–H···N, N–H···O, C–H···Cl and C–H···O hydrogen bonds, lp···π and anion···π^+ interactions (Tables S1, S3). In the first substructure, the protonated pyridine ring nitrogen atom N(1) in the molecules at (x, y, z) acts as donor to the carbonyl oxygen atom O(2) in the molecule at (1/2–x, –1/2+y, 3/2–z) and conversely the amine

nitrogen N(5) acts as donor to the N(3) atom in the molecule at (1/2+x, 1/2+y, z); thus forming a supramolecular network structure in (110) plane (Fig. S3a). In another substructure the Cl(1) anion acts as triple acceptor to the carbon atoms C(1) and C(4) and to the amine nitrogen atom (N5); thus generates another two-dimensional assembly in (110) plane (Fig. S3b). Another contact between the substituted C≡N group of the cationic moiety and the π-system of the B-ring is observed (Fig. S3c) where the uncoordinated N(4) atom in the molecule at (x, y, z) is in contact with the centroids of the B-ring at (1–x, y, 3/2–z) with a separation distance of 3.702(2) Å. Due to the self-complementarity nature, the molecules forms a dimer through lp···π interaction (Fig. S3c). Again, in both sides of the dimeric fragment, the Cl ion is oriented toward the centroid of the protonated pyridine ring and the structure possesses anion···π^+ interaction where the separation distance between the ring centroid and the Cl ion is 3.977(8) Å. Thus, cooperative lp···π and anion···π^+ interactions may increase the strength of the assembly (Fig. S3c).

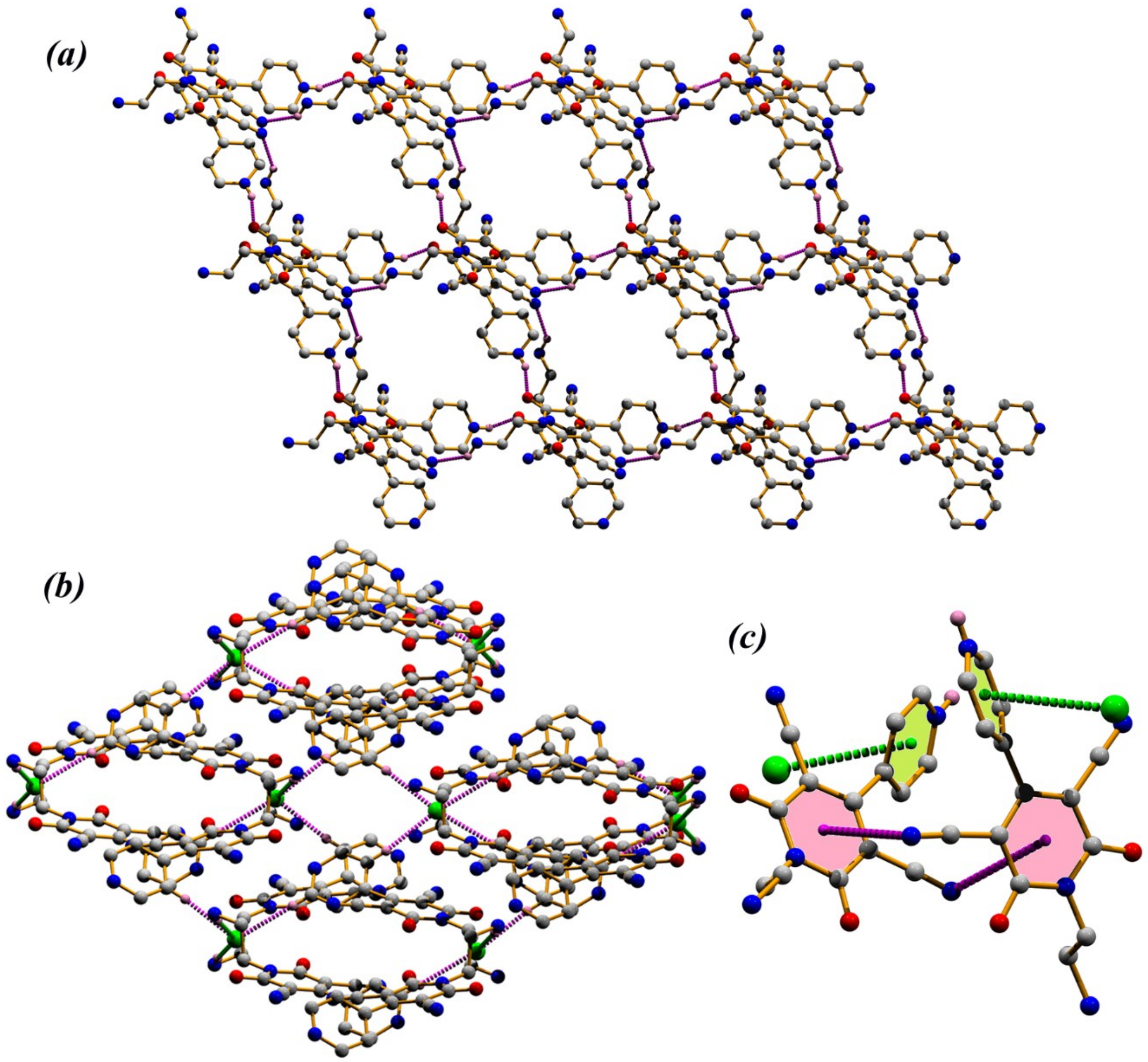

Fig. S3 (a) Supramolecular network generated through N–H···N and N–H···O hydrogen bonds, (b) network through C–H···Cl bonds, (c) lone pair···π and anion···π^+ interactions in **3**.

In asymmetric unit of **4**, comprises of the same cationic moiety, one tetrafluoroborate anion and two solvent water molecules which exhibits N–H···N, N–H···O, O–H···O hydrogen bonds, π···π stacking, lp···π and anion···π^+ interactions (Tables S1–S3). The pyridine ring nitrogen atom N(1) in the molecule at (x, y, z) acts as donor to the nitrogen atom N(3) in the molecule at (2–x, 2–y, 1–z), so generating a centrosymmetric $R_2^2(18)$ dimeric ring centered at (1, 1, ½) (Fig. S4).

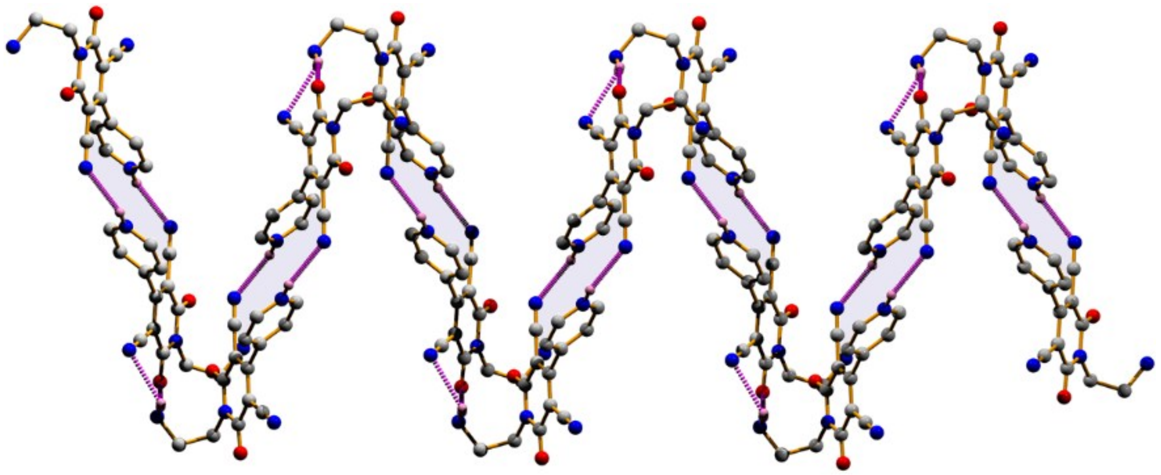

Fig. S4 Zigzag chain propagating along [010] direction in **4**.

Addition reinforcement through N–H···N hydrogen bonding between N(5) and N(4) atoms leads the molecules to generate a zigzag chain along [010] direction (Fig. S4). The parallel stacked zigzag chain are interconnected by the H-bonding between amine N(5) and solvent water oxygen O(3) atom along with an intermolecular H-bonds between water oxygen and carbonyl oxygen atoms; thus a two-dimensional supramolecular network is generated in the (110) plane (Fig. S5a). The interconnection between the amine nitrogen atoms and the role played by solvent water oxygen atoms as donor as well as acceptor leads the molecules to generate a supramolecular layered architecture in (011) plane (Fig. S5b). In another substructure, the interconnection of molecules through π···π stacking, lp···π and anion···π^+ interactions are optimized. Rings (B) of the molecules at (x, y, z) and (1–x, 2–y, 1–z) are strictly parallel, with an interplanar spacing of 3.358 Å, and a ring centroid separation of 3.9639(9) Å, corresponding to a ring offset of 3.29 Å (Fig. S5c). The uncoordinated carbonyl oxygen atom O(2) is oriented toward the π-face of the B-ring of cationic moiety. The distance between O(2) and the centroid of the B-ring is 3.3583(14) Å [C(9)···Cg(B) = 3.4234(17) Å where Cg(B) is the centroid of the B-ring]. This carbonyl oxygen atom O(2) approaches the π-face with an angle 82.56(9)° in the

molecule at (1–x, 2–y, 1–z), therefore reflecting a significant lone pair···π interaction (Fig. S5c). Because of the self-complementary nature, the F(1) atom of one tetrafluoroborate anion in the molecule at (x, y, z) is oriented toward the π-cloud of protonated A-ring in the molecule at (–1+x, y, z) (Fig. S5c), where the separation distance between the ring centroid and the F(1) atom is 3.747(2) Å (Table S3). The shortest separation distance is F(1)···C(2) = 3.529 Å, which indicates anion···π^+ interaction in **4** (Fig. S5c).

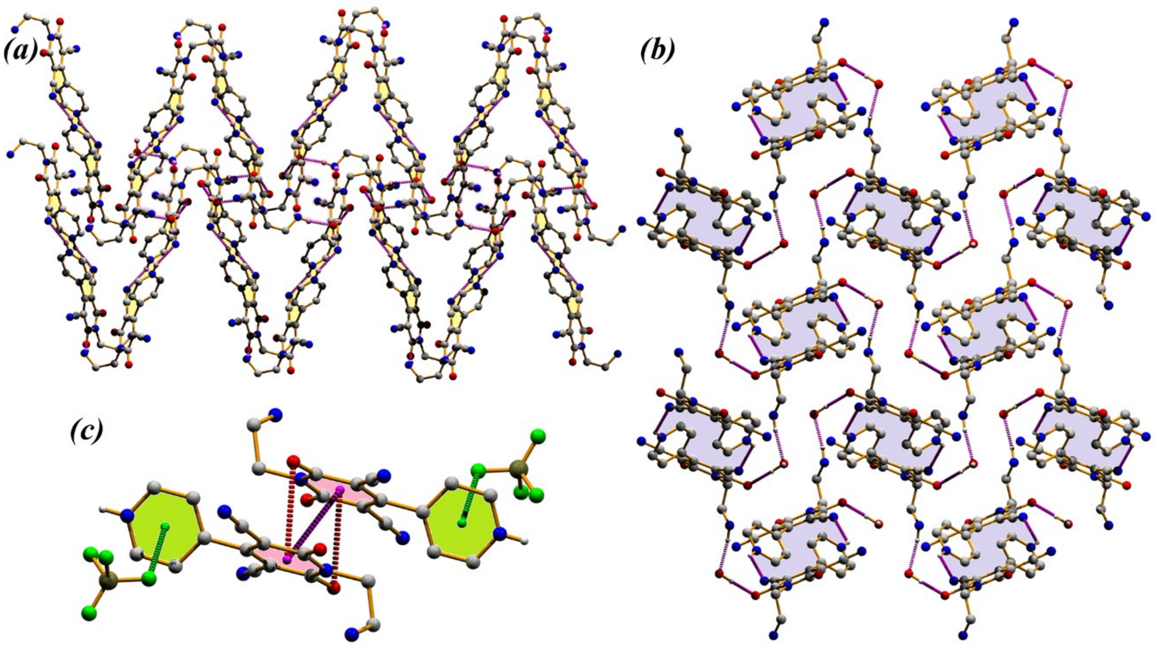

Fig. S5 (a) Formation of supramolecular network in (110) plane, (b) layered network in (011) plane, (c) perspective view of π···π, lp···π and anion···π^+ network in **4**.

Table S1 Geometrical parameters of hydrogen bonds in 2–4

**D–H···A**	**D–H [Å]**	**H···A [Å]**	**D···A [Å]**	**D–H···A**	**Symmetry**
**2**					
N1–H1···O8	0.86	2.22	2.926(3)	140	–
N1–H1···N3	0.86	2.40	3.023(3)	129	2–x, –y, 1–z
N5–H5A···O7	0.89	2.11	2.885(3)	144	x, 1/2–y, 1/2+z
N5–H5A···N4	0.89	2.58	3.051(3)	114	x, 1/2–y, –1/2+z
N5–H5B···O7	0.89	2.00	2.881(3)	171	1–x, 1/2+y, 1/2–z
N5–H5C···O2	0.89	2.06	2.935(2)	169	x, 1/2–y, –1/2+z
O7–H7A···O8	0.83	2.07	2.870(3)	162	2–x, –y, 1–z

O7–H7B···O1	0.84	2.00	2.817(2)	165	–
O8–H8A···N4	0.82	2.49	3.088(3)	131	2–x, –y, 2–z
O8–H8A···O5	0.82	2.33	2.985(7)	137	x, 1/2–y, 1/2+z
O8–H8B···O3	0.83	2.13	2.954(4)	172	2–x, –1/2+y, 3/2–z
C1–H1A···N3	0.93	2.58	3.116(3)	117	2–x, –y, 1–z
C2–H2···O4A	0.93	2.39	3.064(8)	129	2–x, –y, 1–z
C4–H4···N3	0.93	2.52	3.406(3)	158	x, 1/2–y, 1/2+z
C5–H5···O4	0.93	2.43	3.336(6)	165	x, 1/2–y, 1/2+z
C14–H14A···O4	0.97	2.59	3.118(7)	114	–1+x, y, z
**3**					
N1–H1···O2	0.86	1.80	2.625(2)	159	1/2–x, –1/2+y, 3/2–z
N5–H5A···Cl1	0.89	2.23	3.1096(19)	167	1/2–x, 1/2–y, 1–z
N5–H5B···N3	0.89	2.18	2.892(2)	136	1/2+x, 1/2+y, z
N5–H5B···N4	0.89	2.51	3.102(2)	125	1/2+x,1/2–y,–1/2+z
C1–H1A···Cl1	0.93	2.81	3.737(2)	172	1/2+x, –1/2+y, z
C2–H2···O1	0.93	2.37	3.217(3)	151	1–x, –y, 1–z
C4–H4···Cl1	0.93	2.76	3.680(2)	171	–
C13–H13A···O2	0.97	2.33	2.670(2)	100	–
**4**					
N1–H1···O4	0.86	2.23	2.935(2)	139	2–x, 1/2+y, 3/2–z
N1–H1···N3	0.86	2.36	2.993(2)	130	2–x, 2–y, 1–z
O3–H3A···O4	0.92	1.99	2.869(2)	159	x, 3/2–y, –1/2+z
O3–H3B···O1	0.88	1.96	2.8197(18)	164	–
O4–H4A···F3A	0.87	2.34	2.964(6)	129	1–x, 1–y, 1–z
O4–H4A···N4	0.87	2.44	3.101(2)	134	x, 3/2–y, –1/2+z
O4–H4B···F1	1.00	1.86	2.847(3)	169	1+x, y, z
N5–H5A···O2	0.89	2.06	2.9391(19)	170	x, 3/2–y, –1/2+z
N5–H5B···O3	0.89	2.02	2.8903(19)	167	1–x, –1/2+y, 1/2–z
N5–H5C···O3	0.89	2.08	2.8782(19)	150	x, 3/2–y, 1/2+z
N5–H5C···N4	0.89	2.60	3.050(2)	113	x, 3/2–y, –1/2+z
C1–H1A···F2	0.93	2.37	3.286(6)	169	1+x, 3/2–y, 1/2+z
C2–H2···N3	0.93	2.51	3.396(3)	160	x, 3/2–y, 1/2+z
C4–H4···F2A	0.93	2.47	3.045(8)	120	1–x, 2–y, 1–z
C5–H5···N3	0.93	2.59	3.113(3)	116	2–x, 2–y, 1–z
C13–H13A···O1	0.97	2.34	2.709(2)	102	–

Table S2 Geometrical parameters (Å, °) for the π···π Stacking interactions in 2 and 4

Cg(i)···Cg(j)	Cg(i)···Cg(j)[Å]	α (°)	β (°)	γ (°)	Cg(i)–perp[Å]	Cg(j)–perp[Å]	Symmetry
**2**							
Cg(B)···Cg(B)	3.961(2)	0.00	31.1	31.1	3.3917	3.3917	1–x, –y, 1–z
**4**							

Cg(B)···Cg(B)	3.9639(9)	0.00	32.1	32.1	3.3581	3.3581	1–x, 2–y, 1–z

Cg(i) and Cg(j) denotes centroid of ith and j[th] ring respectively. Cg(B) [N2/C6/C7/C8/C9/C10]

Table S3 Geometrical parameters (Å, °) for the anion···π and lone pair···π interactions for the title compounds 2–4

**Y–X(I) ···Cg(J)**	**X···Cg [Å]**	**Y···Cg [Å]**	**Y–X···Cg (°)**	**Symmetry**
**2**				
C(9)–O(2)[1]···Cg(B)	3.380(2)	3.437(2)	82.23(11)	1–x, –y, 1–z
Cl(1)–O(3)[2]···Cg(A)	3.609(3)	4.252(2)	107.84(14)	x, y, z
**3**				
C(12)–N(4)[1] ···Cg(B)	3.702(2)	4.151(2)	105.10(11)	1–x, y, 3/2–z
Cl(2A)a–Cl(2A)[2]···Cg(A)	3.977(8)	5.012(8)	154.3(7)	x, y, z
**4**				
B(1)–F(1)[2]···Cg(A)	3.747(2)	4.322(3)	105.82(15)	–1+x, y, z
C(9)–O(2)[1]···Cg(B)	3.3583(14)	3.4234(17)	82.56(9)	1–x, 2–y, 1–z

Cg(j) denotes centroid of jth ring of the compound. Cg(A) [N1/C1/C2/C3/C4/C5], Cg(B) [N2/C6/C7/C8/C9/C10]

REFERENCES

1. S. K. Seth, I. Saha, C. Estarellas, A. Frontera, T. Kar and S. Mukhopadhyay, *Cryst. Growth Des.*, 2011, **11**, 3250–3265.
2. P. Manna, S. K. Seth, A. Das, J. Hemming, R. Prendergast, M. Helliwell, S. R. Choudhury, A. Frontera and S. Mukhopadhyay, *Inorg. Chem.*, 2012, **51**, 3557–3571.
3. P. Manna, S. K. Seth, A. Bauzá, M. Mitra, S. R. Choudhury, A. Frontera and S. Mukhopadhyay, *Cryst. Growth Des.*, 2014, **14**, 747–755.
4. M. Mitra, P. Manna, A. Bauzá, P. Ballester, S. K. Seth, S. R. Choudhury, A. Frontera and S. Mukhopadhyay, *J. Phys. Chem. B*, 2014, **118**, 14713–14726.

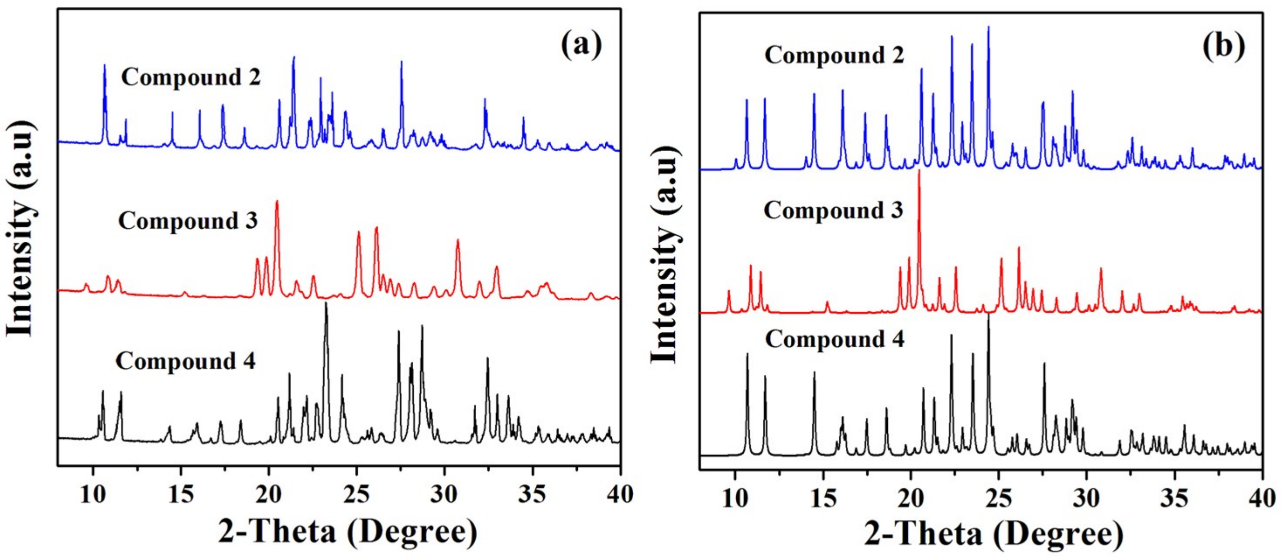

Fig. S6 PXRD patterns of (a) synthesized compounds **2** (blue), **3** (red), and **4** (black), (b) simulated pattern from single crystal data of compounds **2** (blue), **3** (red), and **4** (black). They match reasonably well.

TG experiments were carried out with compounds **2** and **4** where two crystal waters are present in each. It was observed that complete loss of water molecules take place at 120 – 130 °C for **2** and at 130 – 135 °C for **4**. Thereafter, at higher temperatures sequential decompositions of the salts start.

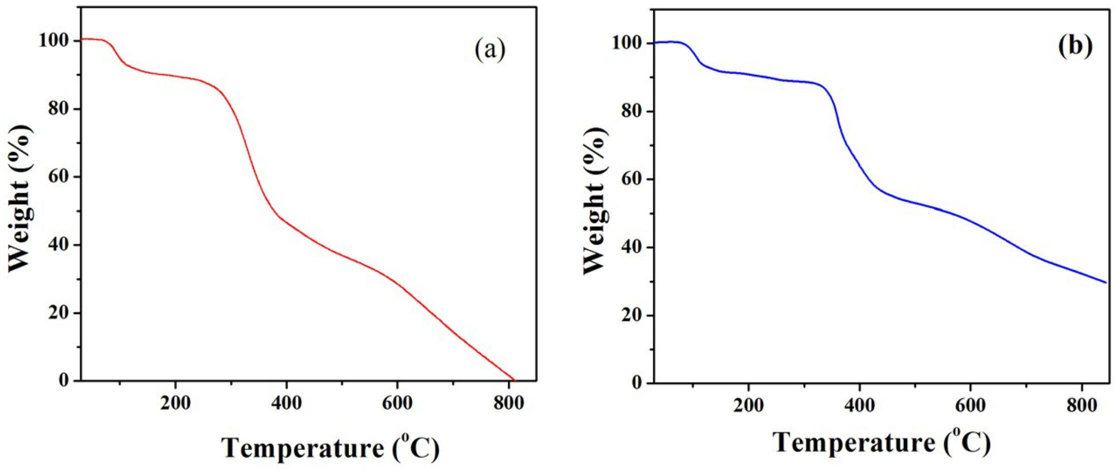

Fig. S7 TGA of (a) compound **2** and (b) compound **4** measured under N_2 atmosphere.

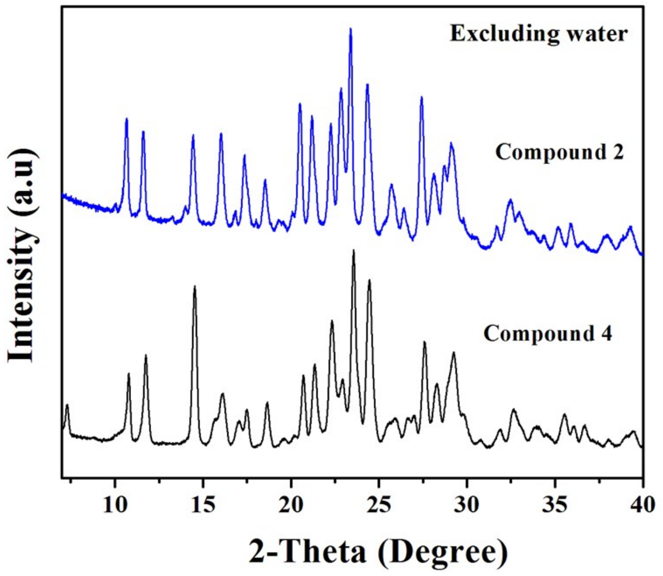

Fig. S8 PXRD patterns of dehydrated compound **2** (blue) and dehydrated compound **4** (black).

A comparison of the PXRD patterns of the dehydrated compounds of **2** and **4** with those of the corresponding hydrated compounds (**Fig. S6 (a))** reveals that both the compounds retained their crystalline nature sufficiently well even after dehydration. The positions of the major diffraction peaks remained almost the same indicating structural similarity of the dehydrated form with its hydrated form. The patterns of the dehydrated compounds get slightly broadened suggesting smaller/nano size of the resulting compound. Due to the associated changes like the absence of a few peaks together with appearance of some new diffraction peaks and variation of relative intensity in regard to the parent (hydrated) phase, it is difficult to comment about the phase structure of the dehydrated forms/compounds.

Table S4 Comparison table showing electrical conductivity data

**Sample Name/Formula**	**Electrical conductivity ($S\ m^{-1}$)**	**Reference**
Cu_3Br(pylimidine-2-thione)$_2$]	6.0×10^{-8}	*Inorg. Chem.*, 2012, **51**, 718–727.
Cu[Ni(2,3-pyrazinedithiolato)$_2$]	1.0×10^{-8}	*Chem. Mater.*, 2010, **22**, 4120–4122.
2,20-((1,4-phenylenebis-(methylene))bis(sulfanediyl))-bis(N-(pyridin-4-ylmethylene)aniline)	7.54×10^{-4}	*Phys. Chem. Chem. Phys.*, 2018, **20**, 24744–24749
(1,4-phenylenebis(azanylylidene)bis-(methanylylidene)bis(2-methoxy-4-phenyldiazenyl)phenol)	14.59×10^{-4}	*New J. Chem.*, 2018, **42**, 13430–13441
1,4-bis-(quinolin-6-yliminomethyl)benzene	9.9×10^{-5}	*J Mater. Sci.*, 2016, **51**, 9394–9403
$C_{14}H_{11}N_5O_2$ (**1**)	1.27×10^{-5}	This work
$C_{14}H_{16}ClN_5O_8$ (**2**)	3.16×10^{-4}	This work
$C_{14}H_{12}ClN_5O_2$ (**3**)	1.68×10^{-3}	This work
$C_{14}H_{16}BF_4N_5O_4$ (**4**)	8.33×10^{-5}	This work

Table S5 Four DNA decamer sequences used for docking study

**Serial no.**	**DNA**	**Sequence**
1	S1	5'-d(GAT<u>GGCC</u>ATC)$_2$
2	S2	5'-d(GAT<u>CCGG</u>ATC)$_2$
3	S3	5'-d(GGC<u>AATT</u>GCC)$_2$
4	S4	5'-d(GGC<u>TTAA</u>GCC)$_2$
